# Fundamentals and Implication of Point of Zero Charge (PZC) Determination for Activated Carbons in Aqueous Electrolytes

**DOI:** 10.1002/advs.202409162

**Published:** 2024-11-13

**Authors:** Sylwia Slesinska, Przemysław Galek, Jakub Menzel, Scott W. Donne, Krzysztof Fic, Anetta Płatek‐Mielczarek

**Affiliations:** ^1^ Poznan University of Technology Institute of Chemistry and Technical Electrochemistry Berdychowo 4 Poznan 60965 Poland; ^2^ Discipline of Chemistry University of Newcastle Callaghan New South Wales 2308 Australia; ^3^ Laboratory for Multiphase Thermofluidics and Surface Nanoengineering Department of Mechanical and Process Engineering ETH Zurich Sonneggstrasse 3 Zurich 8092 Switzerland; ^4^ Unbound Potential GmbH Bönirainstrasse 14 Thalwil 8800 Switzerland

**Keywords:** activated carbon (AC), aqueous electrolyte, electrochemical capacitor (EC), electrochemical quartz crystal microbalance (EQCM), point of zero charge (PZC)

## Abstract

The point of zero charge (PZC) is a crucial parameter for investigating the charge storage mechanisms in energy storage systems at the molecular level. This paper presents findings from three different electrochemical techniques, compared for the first time: cyclic voltammetry (CV), staircase potentio electrochemical impedance spectroscopy (SPEIS), and step potential electrochemical spectroscopy (SPECS), for two activated carbons (ACs) with 0.1 mol L^−1^ aqueous solution of LiNO_3_, Li_2_SO_4_, and KI. The charging process of AC operating in aqueous electrolytes appears as a complex phenomenon – all ionic species take an active part in electric double‐layer formation and the ion‐mixing zone covers a wide potential region. Therefore, the so‐called PZC should not be considered as an absolute one‐point potential value, but rather as a range of zero charge (RZC). SPECS technique is found to be a universal and fast method for determining RZC, as applied here together with the EQCM. In most cases, the RZC covers a potential range from ≈100 to ≈200 mV and the correlation of the range with the carbon microtexture is clear, highlighting the role of the ion‐sieving effect. It is postulated that PZC for porous materials in aqueous electrolytic solutions should be considered instead as RZC.

## Graphical abstract

The point of zero charges (PZC) is investigated using three electrochemical techniques: cyclic voltammetry (CV), staircase potentio electrochemical impedance spectroscopy (SPEIS), and step potential electrochemical spectroscopy (SPECS). Results show that PZC should be considered as a range of zero charge (RZC) for porous materials, with SPECS emerging as a universal method for determining RZC.

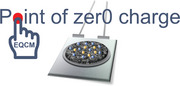

## 1 Introduction

Research and development actions are needed to support the transition to green energy by proposing reliable energy storage systems. In an electrochemical society, there is a continuous discussion of the superiority of devices adapted to specific applications over universal ones. This has triggered the growing demand for more reliable and efficient energy storage devices, such as batteries or electrochemical capacitors (ECs). The latter offers much higher specific power (>10 kW kg^−1^) and cyclability (>10^6^) than current state‐of‐the‐art batteries,^[^ ^1^, ^2^, ^3^, ^4^, ^5^, ^6^ ^]^ but ongoing research is still focused on increasing their energy density while retaining their particular charge/discharge properties. Since energy in ECs is stored mainly through the adsorption of ions at the electrode/electrolyte interface in the electric double‐layer (EDL), ion dynamics investigations are in this context of essential importance. The process efficiency in ECs is largely affected by the electrode properties and ion behavior (both in the bulk of the electrolyte and within the electrode porosity).^[^ ^7^, ^8^ ^]^ However, it is difficult to distinguish these processes on the macroscale.

ECs use highly porous activated carbon (AC) materials,^[^ ^9^, ^10^, ^11^, ^12^, ^13^ ^]^ which are characterized by a well‐developed surface area (>1500 m^2^ g^−1^),^[^ ^14^ ^]^ good electrical conductivity (50 S m^−1^),^[^ ^15^ ^]^ low cost,^[^ ^16^ ^]^ and versatile porosity.^[^ ^17^ ^]^ Although, recent studies have also found that the ion adsorption process in ACs is not straightforward. This process is affected by the ion‐exchange mechanism, ion‐pore size mismatch, and the ion solvation effect;^[^ ^18^ ^]^ these all have an impact on the performance of the carbon electrode and need to be considered. For this purpose, a wide range of advanced in situ techniques have been recently explored, such as electrochemical quartz crystal microbalance (EQCM),^[^ ^7^, ^18^, ^19^, ^20^ ^]^ nuclear magnetic resonance spectroscopy (NMR),^[^ ^21^, ^22^, ^23^, ^24^ ^]^ infrared spectroscopy (IR),^[^ ^5^ ^]^ and electrochemical dilatometry (ECD),^[^ ^25^, ^26^ ^]^ with the ability to elucidate the charge storage mechanisms directly at the nanoscale.

The selection of an electrolyte for ECs depends on the application and three main electrolyte groups can be distinguished: aqueous, organic, and ionic liquid.^[^ ^27^ ^]^ H_2_SO_4_ or KOH are used in ECs due to their high ionic conductivity, as well as serving as a good and well‐understood environment for the fundamental characterization of new materials.^[^ ^28^, ^29^ ^]^ Inorganic salts have emerged as an alternative to highly corrosive alkaline and oxidative acidic electrolytes. Due to their nearly neutral pH in aqueous solutions, they exhibit overpotentials for oxygen (OER) and hydrogen (HER) evolution reactions, which extend the operational voltage window beyond thermodynamic stability.^[^ ^30^ ^]^ Today, more insights into the performance of neutral aqueous electrolytes are provided to understand their limitations and push their operation. Molecular level dynamics of sulfate‐based,^[^ ^31^, ^32^ ^]^ and iodide‐based systems^[^ ^8^, ^33^ ^]^ have been successfully described. The description of the charging mechanism for nitrate‐based or other neutral electrolytes is still sought, despite their common use in primary tests for new materials.^[^ ^34^, ^35^ ^]^

EQCM enables the electrode/electrolyte interface to be monitored at the molecular level with advanced in situ tracking of viscoelastic property changes.^[^ ^1^, ^7^, ^36^, ^37^, ^38^, ^39^, ^40^ ^]^ For EQCM measurements, the correct determination of the point of zero charge (PZC) is crucial because it determines the electrode potential value at which the formation of the EDL does not require any additional charge in a specific environment. Moreover, it influences all subsequent electrosorption processes at the electrode/electrolyte interface and describes the ion‐sieving effect.^[^ ^41^, ^42^ ^]^ In this way, when the electrode is polarized outside the PZC, it will either attract cations or anions, or repel/reorganize specifically adsorbed ions from the electrode surface. EQCM enables the direct determination of the type of process (adsorption or desorption) and can help to provide a description of the kind of species adsorbed. However, detailed description and direct detection of ions concentration at the electrode/electrolyte interface can be done only using the NMR technique. For planar (formally non‐porous) stainless steel electrodes, it has been recently shown that the hydration shell of the physiosorbed species in the EDL shifts the PZC.^[^ ^43^, ^44^ ^]^ In addition, the PZC of metal surfaces is a pH‐independent variable, tested for dilute aqueous solutions; nevertheless, pH influences other reaction potentials, as they might be affected by solution species like H^+^.^[^ ^44^ ^]^ When porous carbon is applied as an electrode, the observed phenomenon cannot be simply discussed, and thus PZC is one of the key parameters to enable reliable data analysis.^[^ ^39^, ^45^, ^46^, ^47^ ^]^

The PZC in electrochemical systems can be evaluated using various techniques. The most common are cyclic voltammetry (CV) and impedance spectroscopy (EIS). Minimum current response in CV tests using high scan rates for organic electrolytes, i.e., 10 mV s^−1[^ ^38^, ^48^ ^]^ can be also verified with in situ measurement of minimal electronic conductivity versus applied potential.^[^ ^49^ ^]^ For organic‐based solution the minimum current versus applied potential is easily detectable. Interestingly, reported cyclic voltammetry for different ECs was not always conducted directly in 3‐electrode EQCM cell, but often in 2‐electrode with reference Swagelok cell.^[^ ^36^, ^50^ ^]^ EIS tests are performed at different potentials (PEIS), allowing one to present specific capacitance versus potential and to find its minimal value.^[^ ^39^, ^46^, ^51^ ^]^ Recently, step potential electrochemical spectroscopy (SPECS) was first reported to be applied to determine PZC.^[^ ^47^ ^]^ Each literature study applies one technique for the determination of PZC, and we find the description of the experimental conditions used very scarce. No clear evidence for a reason or purpose for application of one of these techniques can be found in the literature up to date.

Besides, SPECS and EQCM were already coupled to investigate the manganese dioxide electrodeposition process as a probe of capacitive behavior.^[^ ^52^ ^]^ However, that study was not focused on PZC determination.

In our study, we explored various techniques that could enable an accurate and fast determination of PZC (with minimal over‐ or underestimation of the PZC value). A set of comparative PZC data was obtained on the basis of three techniques: staircase potentio electrochemical impedance spectroscopy (SPEIS), CV, and SPECS, all recorded directly in an EQCM setup, to create a rational comparison among the techniques described in the literature. The PZC value determined in the EQCM cell was compared with the value obtained in the 2‐electrode with reference electrode Swagelok system and the 3‐electrode cell, so‐called volume cell, to show the influence of cell design on the PZC value. We also reported changes in EQCM responses for the microporous carbon electrode in three different aqueous electrolytes (sulphate, nitrate, and iodide based) to represent specific EDL formation at the porous carbon interface in the presence of water molecules despite the presence of polarized species. The water molecules, due to their concentration in the solution (0.1 mol L^−1^ inorganic salt electrolytic solutions were tested) extend PZC to a region of zero charge, further denoted as RZC. To confirm the observed phenomena, two microporous carbons and a planar stainless‐steel surface were tested with three selected aqueous electrolytic solutions.

## 2 Results and Discussion

### 2.1 Point of Zero Charge Determination

To study porous activated carbons with a non‐uniform and wide pore size distribution, dilute aqueous solutions (0.1 mol L^−1^) were selected to keep the electroactive species concentration at a level that does not exceed the detection limits of the EQCM used (measurable frequency change). To ensure the high applicability of our study, the nonideal electrode material, with a significant micropore volume, *V_micro_* > 0.64 cm^3^ g^−1^, was tested**—**representing activated carbons used in the full cell studies and possible commercial devices (Figures S1 and S2, Table S1, Supporting Information).

**Figure 1:**
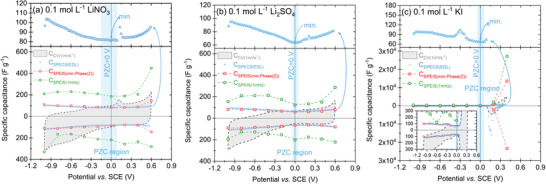
Specific capacitance versus potential calculated from three electrochemical techniques: CV (gray shade), SPECS (blue triangular scatter), and SPEIS (red square and green circular scatter) for YP‐50F and 0.1 mol L^−1^ electrolytic solutions in EQCM cell a) LiNO_3_, b) Li_2_SO_4_, and c) KI. The upper part of the plot represents the zoomed SPECS‐specific capacitance versus potential in the *E_min_* to *E_max_* direction.

In **Figure** **1**, the PZC values obtained by the CV, SPECS and SPEIS techniques in the EQCM cell are compared for 0.1 mol L^−1^ LiNO_3_ (Figure 1a), Li_2_SO_4_ (Figure 1b) and KI (Figure 1c) with the YP‐50F electrode material on the specific capacitance versus potential plot (for detailed CV – Figure S7, SPEIS – Figure S8, and SPECS methodology, see Supporting Information). Aqueous electrolytes were selected due to their satisfactory electrochemical performance at high voltages, >1.2 V, and satisfactory long‐term stability.^[^ ^8^, ^31^, ^34^ ^]^

The experimental conditions for CV (for 5 and 50 mV s^−1^, see Figure S7, Supporting Information) and SPECS were convergent, and the currents recorded from these techniques were converted to specific capacitance. For CV, 1 mV s^−1^ was applied and for SPECS, *E* = 10 mV with *t* = 10 s, providing an average scan rate of 1 mV s^−1^. The PZC is determined as the lowest capacitance (current) value^[^ ^53^ ^]^ in the intermediate potential range (directly correlated with the capacitance (*C_CV_*) calculated from the CV technique based on Equation S2, Supporting Information). However, this method might not be fully accurate since the voltammetry response is derived from the full current response, which is also related to diffusion‐limited processes and EDL formation. The PZC corresponds to an electrostatic interaction between the electrode and the electrolyte (EDL formation only). This process might not be fast enough, particularly in a porous medium, such as activated carbons, being affected by transport‐related phenomena. Diffusion‐limited processes include those related to the oxidation/reduction of compounds and the intercalation/insertion and residual processes resulting from side reactions (such as electrolyte decomposition).^[^ ^54^ ^]^ The Faradaic current could superimpose the EDL response, as shown in the CV profiles (dashed black lines in Figure 1a–c) near 0.15 V vs. SCE for LiNO_3_, 0.15 and 0.2 V vs. SCE for Li_2_SO_4_ and −0.3 V vs. SCE for KI. This redox peak could be attributed to a redox response from quinone/hydroquinone‐type functionalities, grafted onto carbon surface.^[^ ^55^ ^]^ Further, as extreme potentials (*E_max_* and *E_min_*) were approached, the box‐shaped CV profile was deformed by the increase in current related to the decomposition of the electrolyte.

*C_SPEIS(1mHz)_* curves for the LiNO_3_‐based system are not symmetric for both polarization directions (see Figure S8, Supporting Information). The same situation occurs for systems with Li_2_SO_4_ (Figure 1b) and KI (Figure 1c). Furthermore, for the KI redox system, the measurement toward *E_min_* was impossible to perform correctly due to the high redox activity in the positive potential values (> 0 V vs SCE).

Unlike CV (Figure S7, Supporting Information) and SPEIS (Figure S8, Supporting Information), which are more prone to experimental limitations, SPECS is determined to be a promising technique for PZC identification, regardless of the experiment conditions (Figure 1; blue squares); thus, universal one for electrochemical capacitor testing. The advantage of this technique includes a short implementation step time (in this case, 1 mV s^−1^). Moreover, the potential shift is quite gentle and enables detailed data to be recorded in the entire potential range, increasing the resolution and accuracy of the recorded data. These potential steps lead to a smooth behavior of (tentative) redox reactions and balanced ion redistribution in the pores. The advantage of SPECS over CV could be also seen in the ability to quantify the capacitive contribution of different charge‐storing mechanisms, including those associated with EDL formation (see Supporting Information).^[^ ^53^, ^54^ ^]^

The PZC in Figure 1 is not a specific potential value for the ACs, with a clear inflection point on the capacitance curve. Here, one can distinguish a wide potential range of RZC (blue region) from −0.15 to 0.09 V vs. SCE, with a comparable capacitance (82 F g^−1^) for the LiNO_3_‐based system (with ±2% variation in the minimal specific capacitance value), providing a PZC range of 240 mV. For Li_2_SO_4_, this region is from −0.04 to 0.05 V (PZC range of 90 mV, data for two concentrations, 0.1 and 1 mol L^−1^, can be found in Figure S9, Supporting Information) and for KI, this region is from −0.05 to 0.06 V (PZC range of 110 mV).

We propose to determine exact PZC values (if needed) in the middle (median) of this low capacitance region, here at 0 V versus SCE for three tested aqueous electrolytic solutions. Interestingly, an increase in *C_SPECS(EDL)_* and total capacitance can also be observed in the potential region where redox processes occur (for all tested electrolytes). Because redox processes induce charge transfer with the specific adsorbed ions, they affect the formation of the EDL itself. Furthermore, this response cannot be differentiated from *C_EDL._* An ambiguous increase in recorded current is observed due to quinone/hydroquinone redox activity of the electrode surface.

**Figure 2:**
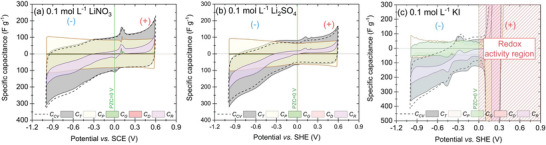
Capacitance components were calculated from the SPECS technique for 0.1 mol L^−1^ aqueous solution of a) LiNO_3_, b) Li_2_SO_4_, and c) KI.

The SPECS technique provides insight into the charging mechanism of electrochemical capacitors by differentiating the capacitance contribution (see Supporting Information for detailed information about the technique and calculations), as shown in **Figure** **2**.

The calculated total capacitance (*C_T_*) of the system is identical to the *C_CV_* (Figure 2) when identical measurement conditions are maintained (in this case, the average scanning rate of 1 mV s^−1^). As expected for porous carbon materials, the main contribution to *C_T_* is made by porous capacitance (*C_P_*) due to the strongly developed specific surface area of the AC used (Figure S1, Supporting Information). The *C_P_* curves show a butterfly shape that is usually visible for organic systems,^[^ ^33^, ^50^ ^]^ and fast repolarization processes are shown in the box‐like shape. The share of geometric capacitance (*C_G_*) is significantly lower for capacitive systems, that is, LiNO_3_ (Figure 2a) and Li_2_SO_4_ (Figure 2b), resulting from the poorly developed electrode surface in direct contact with the electrolyte (low ratio of the geometric electrode interface to the electrolyte volume). Interestingly, for the redox‐based system (Figure 2c), *C_P_* and *C_G_* are almost equal in the potential range lower than PZC, i.e., in the capacitive region, due to specific iodide adsorption onto the carbon surface. Unfortunately, the computational method does not allow to make such predictions (calculations of *C_P_* and *C_G_*) for the redox active region; thus, it was not applied. In the systems studied, the smallest capacitive contribution comes from ion diffusion (*C_D_*), showing that the main charge storage mechanism is based on EDL formation. Moreover, residual capacitance (*C_R_*) is responsible for any discrepancies from the ideal box‐shaped shape characteristic for a capacitive response; the value of *C_R_* increases in the potential regions where the electrolyte decomposition, specific ion adsorption, or redox processes occur. *C_R_* is responsible for the overall *C_T_* cyclic voltammogram shape deviating from the ideal *C_P_* capacitive curve.

The influence of the potential range was also compared (1 vs. 1.6 V) on the PZC range for 0.1 mol L^−1^ LiNO_3_. The same experimental conditions were applied in the narrow and wide potential tests using the SPECS technique. CV computed for both potential ranges do not differ qualitatively (Figure S9, Supporting Information). Both cyclic voltammograms represent capacitive behavior, visible in a rectangular‐like shape. RZC differs here by 50 mV (Table S6, Supporting Information). This negligible difference results from using fresh experimental cells for this study. Thus, all components: carbon, electrode coating, electrolyte volume, inorganic salt impurities, etc. affect the result. Although, it was proved that the wide voltage window, i.e., 1.6 V, for selected experimental conditions (carbon and aqueous electrolyte) is still stable and reliable.

Our study also included the influence of cell construction on PZC values (see Figure S10, Supporting Information), as various experimental approaches are used in the literature. Unfortunately, a potential shift in electrochemical behavior was observed (Figure S10, Supporting Information) and resulted from different uncompensated resistance factors (Figure S11, Supporting Information). In addition, it seems that tested reference electrodes might influence the determination of the PZC value. It has been proven, that in contrast to overall cell construction (volume of electrolyte, working electrode loading, the distance between electrodes, etc.), reference electrode has a negligible influence (Figure S13, Supporting Information); thus, for short‐term experiments, no Cl^−^ migration from the SCE electrode was tracked. The electrochemical tests of neutral‐pH aqueous ECs were not susceptible to the RE type. However, cell construction had a large impact on individual electrode stability and should be considered while determining PZC.

**Figure 3:**
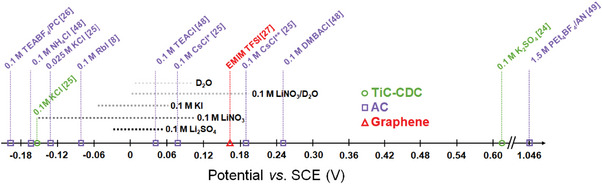
Summary of the PZC values reported in the literature and the RZC presented in our work for various aqueous and organic solutions; all recalculated versus SCE. Differentiation of the electrode material is as follows: Ti‐CDC (circular scatter), AC (square scatter), and graphene (triangular scatter).^[^ ^50^, ^56^ ^]^

In particular, all tested aqueous solutions are characterized by a wide PZC potential range (**Figure** **3**) when determined using the SPECS method (min. capacitance value ±1% or ±2%, please see Table S5, Supporting Information). The LiNO_3_‐based system shows mainly capacitive charge storage behavior^[^ ^34^, ^35^ ^]^ and also has the widest potential PZC range (≈240 mV). Thus, the region of ion reorganization/mixing is not limited by the potential value itself, and both the Li^+^ cations and the NO_3_^−^ anions have the same affinity for the electrode. On the contrary, KI is a pure redox system^[^ ^56^, ^57^, ^58^, ^59^, ^60^ ^]^ characterized by a narrow PZC potential range (110 mV). Beyond this narrow potential range, other mechanisms occur, such as specific ion adsorption on the electrode surface.^[^ ^8^ ^]^ Considering these facts and the ambiguous Li_2_SO_4_ charge storage mechanism,^[^ ^31^ ^]^ it can be stated that it is a pseudocapacitive electrolyte. Its PZC range is rather narrow (90 mV), which can indicate SO_4_^2−^ specific adsorption on the electrode surface. Additionally, the SO_4_^2−^ ion flux during positive electrode charging has thus far been excluded.^[^ ^31^, ^32^ ^]^

The parameter that should also be carefully considered when determining PZC is the mass loading of the electrode (Figure S4, Supporting Information). Higher mass loading increases capacitance by providing more active sites for charge storage. Lower mass loading reduces available ion interaction sites, impacting the clear observation of the RZC. In porous materials, mass loading influences ion penetration (diffusion) into the pores and EDL formation. Thicker electrodes may experience uneven charge distribution, poor conductivity, and potential gradients, complicating the determination of RZC. Additionally, higher mass loading can enhance Faradaic processes, further obscuring the identification of the zero potential range. It is also necessary to remember about the limitation in the EQCM system – the frequency range. Too large of an electrode mass or too large of the frequency changes during experiments (e.g., caused by a large mass of the ions considered or too high electrolyte concentration) may result in the output of the registered signal within the previously selected frequency region. Therefore, 2‐electrode systems and 3‐electrode system, as well as, full cell studies and fundamental ones, should be characterized independently with great caution to the limiting factor for observed phenomena.

The determination of the RZC region in strong oxidation‐reduction systems can become less straightforward than in pure EDL systems. In a simple electrochemical system without faradaic processes (no redox reactions), the RZC reflects the electrostatic balance between the electrode and the electrolyte. In a system with strong redox reactions, the RZC may shift depending on the redox couples present and the pH change. Redox‐active species in the electrolyte might interact with the electrode in a way that alters the effective surface charge (via specific adsorption).^[^ ^8^ ^]^ These redox reactions influence the electrode potential, and it might be more appropriate to consider the system's overall electrochemical behavior rather than isolating the RZC. The RZC still represents the minimal electrostatic interaction with the surrounding electrolytic solution, but the redox processes cause dynamic charging conditions. In systems where the material exhibits strong redox properties and has a well‐developed specific surface area, determining the RZC is still feasible (example: KI as electrolytic solution Figure 1c). The highly developed surface of the material allows for a more precise determination of the RZC due to the distinct appearance of the minimum capacitance, which increases with either higher or lower applied potential (*E_max_* and *E_min_*). This is due to ion penetration into the pores and the expansion of the EDL. Conversely, for materials with less developed specific surfaces, determining the RZC may be more challenging (Figure S14, Supporting Information).

The width of RZC seems to also correlate to the size and charge of the ion present in the electrolytic solution. As shown in **Table** **1** below, SO_4_^2−^ has not only a larger ionic radius, but also a higher charge compared to I^−^ and NO_3_^−^ anions. This favors stronger electrostatic attraction toward the oppositely charged surface of the carbon electrode, resulting in increased specific adsorption, creating a net charge on the electrode surface. Consequently, only a small change in the electrode potential is necessary to affect this state and push it out of the equilibrium state, resulting in a narrower RZC.

**Table 1:** Ionic radius and RZC for anions in electrolytes used in this research.

Electrolyte	Anion	Ionic radius^[^ ^61^, ^62^ ^]^	RZC
0.1 m Li_2_SO_4_	SO_4_^2−^	0.242 nm	90 mV
0.1 m LiNO_3_	NO_3_^−^	0.177 nm	240 mV
0.1 m KI	I^−^	0.216 nm	110 mV

We also believe that the effect of concentration on RZC needed to be verified for further clarification, that's why we have tested 1 and 0.1 mol L^−1^ Li_2_SO_4_ (Figure S9 and Tables S5 and S6, Supporting Information). Most of the low‐concentration RZC overlap with the high concentration solution. However, the RZC region is highly extended due to a higher number of charges in the solution able to take part in EDL formation. Thus, the higher the concentration, the wider the RZC region. This also indicates that each system should be treated individually, and each studied concentration offers additional information for other researchers.

Studies on PZC determination methods are very limited in the literature; however, the authors usually refer to one specific measurement/technique; a minimum conductance value or a minimum capacitance value. At the PZC, the net charge on the electrode surface is zero, and the EDL capacitance reaches a minimum. Direct correlation lies in the fact that the minimum capacitance directly corresponds to the point where the net charge on the electrode surface is zero. Thus, the minimum capacitance is often used as a reliable indicator of the PZC, as it directly reflects the point of zero net charge on the electrode surface. Similarly, at the PZC, the electrode surface has no net charge, and the double layer is relatively symmetrical, which can reduce the ease of ion transport.

These values, Figure 3, were obtained by different techniques, varying in the experimental setups and experimental electrochemical conditions. For the ACs studied, YP‐50F and DLC30 (Figure S15, Supporting Information), the wide PZC region is believed to result from the electrolyte/electrode interactions and a high share of solvent molecules during EDL formation.^[^ ^64^ ^]^ AC has a wide variety of surface functional groups, high tortuosity, 3D porosity, and specific structure (Figures S1 and S4, Tables S1, S2, S3, Supporting Information). The planar metallic electrode (without defects and with a low specific surface area) correlates to a wide PZC region (Figure S14, Supporting Information), such as a wide potential window for ionic liquid on glassy carbon electrodes. For the two ACs studied, the PZC region is much narrower than that for the planar resonator (Figure S15, Supporting Information). However, for materials studied with D_2_O, no difference in the RZC range is observed, confirming a particular electrolyte ion/electrode interaction when an inorganic salt is present.

### 2.2 EQCM Data Evaluation

**Figure 4:**
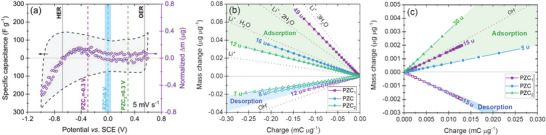
EQCM characterization of 0.1 mol L^−1^ Li_2_SO_4_ and YP‐50F: a) specific capacitance versus potential at 5 mV s^−1^ with the assigned PZC; b) mass:charge plot for the assigned PZC in the polarization toward lower potential values from the PZC; c) mass:charge plot for the assigned PZC in the polarization toward higher potential values from the PZC.

The previous section addressed the proper and accurate determination of the PZC. However, in addition to the determination of PZC for 0.1 mol L^−1^ LiNO_3_, Li_2_SO_4_, KI, and 0.1 mol L^−1^ LiNO_3_ in D_2_O (Figure 3), the significance of these data has not yet been quantified. Therefore, basic electrochemical tests (cyclic voltammetry at 5 mV s^−1^) in combination with EQCM were performed to study the ion flux on the carbon electrode during the charging process (validation of the experimental setup is presented in Figure S16, Supporting Information). To interpret the influence of the PZC value on the evaluation of the data, a Li_2_SO_4_‐based system was selected, and the data is presented in **Figure** **4**. This system is: i) broadly described in the literature, ii) has a complex charging mechanism, and iii) has promising electrochemical performance (very long cyclability).^[^ ^31^ ^]^

After determining PZC using the techniques mentioned (average PZC = 0 V), mass change calculations were performed to establish PZC and two other artificially imposed values (PZC_1_ and PZC_2_). This results in a recalculation where the PZC is the following: 1) ‐300 mV from PZC (PZC_1_) and resembles the PZC determined in the Swagelok cell (Figure S11, Supporting Information), 2) PZC = 0 V vs. SCE and is considered as the correct value determined for the studied system (Figure 1) and 3) +300 mV from PZC (PZC_2_). In total, this provides a wide range of potential *ΔE* = 600 mV in which PZC is considered, imposed, and discussed.

In Figure 4a, the hydrogen evolution potential (HER) is shown; the HER of this electrolyte equals −0.681 V vs. SCE, and the oxygen evolution potential (OER) of this electrolyte equals +0.549 V vs. SCE. According to Faraday's law (Equation 1), the change in mass (related to dissolution or deposition) is linearly correlated with the amount of charge that passes through the electrochemical setup. From the slopes of the mass:charge curves (Figure 4b,c), both adsorption and desorption data show multistep processes ongoing at the electrode/electrolyte interface (determination of ionic species is done based on Table S8, Supporting Information). According to the literature, one can assume the adsorption of Li^+^ as well as its solvated forms, i.e., Li^+^ ∙ 2H_2_O (with *Δm* = 42.97 u) and OH^−^ (with *Δm* = 17.01 u), during negative and positive polarizations, respectively (Table S8, Supporting Information).^[^ ^31^ ^]^ This counter‐ion adsorption/desorption process is considered as the amount of positively and negatively charged species in PZC can be equal resulting in a net zero charge value. In consequence, Figure 4b shows that the adsorption process is followed by the desorption process. In Figure 4c a two‐step process is observed only for PZC_1_, with an initial desorption and then adsorption of the mass difference (15 u). For negative polarization, the ionic species with a molecular weight between Li^+^ and hydrated Li^+^ ∙ 3H_2_O are responsible for the mass change. However, for positive polarization, where SO_4_^2−^ or OH^−^ are anticipated, none of these were found. Therefore, the processes that occur at the positive electrode/electrolyte interface are not related to single ion adsorption. This is potentially related to: the carbon oxidation process that can continuously occur, specific SO_4_^2−^ ion adsorption on the YP‐50F electrode, SO_4_^2−^ redox reactions (to S^2+^ and/or S^4+^ oxidation states^[^ ^31^ ^]^), or ion reorganization. Thus, the matching of ion flux for sulfate‐based electrolytes is not that straightforward.

**Figure 5:**
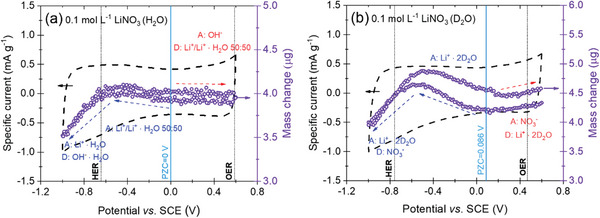
The charging mechanism for 0.1 mol L^−1^ LiNO_3_ and YP‐50F presented in specific capacitance versus potential at 5 mV s^−1^ in a) H_2_O and b) D_2_O.

The electrolytic solution of 0.1 mol L^−1^ LiNO_3_ was studied using EQCM for the first time (**Figure** **5**). This electrolyte has been reported as a capacitive one (even at low concentrations);^[^ ^35^ ^]^ therefore, ion fluxes based on Li^+^ and NO_3_^−^ were anticipated.

Since the negative polarization range was largely extended from the PZC in all measurements, LiNO_3_‐based systems display adsorption followed by desorption while approaching increasingly lower potential values. When the 0.1 mol L^−1^ solution was prepared in DI water, no NO_3_^−^ anion flux was detected (Figure 5a), which led to the conclusion that the NO_3_^−^ anion, like SO_4_^2−^, was more prone to redox processes than EDL formation. It seems that NO_3_^−^ is likely to be reduced (to NO_2_ and/or NO), due to the oxidation of carbon surface – leading to the red‐ox balance and equilibrium at the interface. It is noteworthy that these gases have been found in another study with on‐line mass spectrometry.^[^ ^64^ ^]^ However, the same solution was prepared using D_2_O (Figure 5b) – characterized by a higher molecular weight (see Supporting Information). In the second case, NO_3_^−^ adsorption and desorption were recorded, accompanied by an opposite sorption process of solvated cation (Li^+^ ∙ 2D_2_O). RZC in this case shifts toward more positive potential values, slightly narrowing its potential range (Figure 3) related to a lower number of NO_3_^−^ ions specifically adsorbed on the electrode surface in the presence of D_2_O molecules instead of H_2_O. The solvation shell of a lithium cation seems to be reasonable at this concentration, and interestingly, when D_2_O is a solvent molecule**—**the solvated Li^+^ ions detected by EQCM have a stable solvation shell. Moreover, a good correlation between the PZC values determined via SPECS and SPEIS and the mass change profile recorded by EQCM can be observed. This result further confirmed the need for accurate PZC determination and its insight into the charging mechanism; dividing the mass change curve correctly into different regimes where either cation or anion adsorption is the leading process in EDL formation. These experiments confirmed that the nitrate‐based electrolyte is capacitive, NO_3_^−^ ions tend to be specifically adsorbed onto the AC surface, and that the flux of NO_3_^−^ ions can be detected for the case where specific adsorption of nitrate anion does not occur. When H_2_O was used as a solvent, the OH^−^ anion was more likely to form EDL than NO_3_^−^. These results also showed that pH, conductivity, ionic species (type and their activity), and solvent molecules influenced the charging mechanism in aqueous‐based EC, emphasizing the importance of considering each system individually, quite often in not a straightforward way.

## 3 Conclusion

The PZC was determined using SPECS, SPEIS, and CV techniques for water‐based solutions. Moreover, the PZC meaning for the description of the charging mechanism for aqueous electrochemical capacitors exploiting AC electrodes was discussed. We proposed a universal method for PZC determination in the EQCM cell using the SPECS technique, leading to the most accurate results, as demonstrated for sulfate‐ and nitrate‐based systems. This revealed their charge mechanisms, which would not have been possible using other PZC determination techniques. We demonstrated that the PZC should be considered as an RZC rather than one value for aqueous electrolytic solutions with activated carbon electrodes, in contrast to organic‐based ECs or ECs using well‐organized electrode materials (homogenous in structure and texture).

We have proposed a guideline for future EQCM studies on charge storage mechanisms in energy storage systems. Regardless of the method selected, the RZC should be directly determined in the EQCM cell. The SPECS technique is reported to be an alternative and comprehensive method to determine the RZC. In addition, this method provides important insights into the charging mechanism via deconvoluting the total specific capacitance into pore‐ and surface‐related ones. The SPEIS experiment can also be useful for the determination of PZC, but the overall prolonged time needed to carry out this experiment can negatively impact the electrolyte stability, especially in the EQCM setup. It is crucial here to consider the specific capacitance calculated for the frequency determined from the Bode plot (close to phase angle −90°) to obtain the most reliable values. In our opinion, the most widely used CV technique cannot be recommended for the determination of RZC because porous activated carbons with a developed surface area (> 1500 m^2^ g^−1^) cause excessive formation of an electric double layer, which obscures the potential at which the minimum charge specifically accumulates at the electrode/electrolyte interface.

Studies of the electrolytic solution of LiNO_3_ show that neutral, polar solvent molecules (H_2_O vs. D_2_O) do not significantly influence the PZC region but quantitatively influence the EDL at the electrode/electrolyte interface – affecting the charge storage mechanism. In aqueous solutions NO_3_^−^ anion is specifically adsorbed onto carbon surface, like SO_4_^2−^—proven in this study, and I^−^. However, the number of species included in this phenomenon can be further analyzed via the application of a different solvent, which was successfully proven with D_2_O solutions.

This systematic and rational study can serve as an indication of the correct determination of RZC and its key importance in energy storage devices. The most valuable take‐home message from our study is to treat each system individually with respect to the selected method and technique used. The principles and limitations of all techniques selected must always be obeyed. Although aqueous‐based systems reveal some similarities, their micro‐ and nanoscale observations show drastic differences in their charge storage mechanism, depending on the active material used, therefore, special attention should be paid to the interpretation of the data from this medium.

## 4 Experimental Section

### Electrodes

Electrodes (coated on a quartz crystal resonator) were prepared based on two microporous ACs – Kuraray YP‐50F (Japan) and Norit DLC Supra 30 (Cabot Carbone S.A.S.; France), denoted as YP‐50F and DLC30, respectively. Their physicochemical characterization is shown in the Supporting Information (Figures S1, S2, and Table S1, S2, Supporting Information). The texture data of the ACs are presented in Figure S1 and Table S1 (Supporting Information). The surface areas of both ACs are similar with values of 1702 and 1780 m^2^ g^−1^ for YP‐50F and DLC30, respectively. Although the microporosity volume (*V_micro_*) is similar at 0.65 cm^3^ g^−1^, the mesopore volume (*V_meso_*) is three times higher for DLC30 (0.25 cm^3^ g^−1^) than for YP‐50F (0.08 cm^3^ g^−1^). Both materials consist mostly of micropores with an average diameter of 0.65 and 0.69 nm for YP‐50F and DLC30, respectively. The powder of one of the ACs and a 5 wt.% solution of poly(vinylidenedifluoride) (PVDF) (Sigma‒Aldrich; USA) in N‐methyl‐2‐pyrrolidone (NMP) (Sigma‒Aldrich; USA) were mixed – please refer to Supporting Information for a schematic of the resonator coating procedure, Figure S3 (Supporting Information). The AC:binder mass ratio in the final electrode material was 80:20. The electrode slurry was then mixed with a magnetic stirrer for 2 h. Stainless steel (SUS304) was used as the current collector of the quartz resonator with a standard finish (9 MHz; 21 mm^2^; SEIKO EG & G; Japan). The collectors were lightly sanded with fine‐grain sandpaper prior to drop casting of the electrode material. Mechanical treatment increases the adhesion of the conductive glue and electrode material to its surface and reduces resistance. After surface cleaning (with acetone), the collector was covered with a thin layer of conductive glue ≈60 µg (0.285 mg cm^−2^; DAG; Henkel). The resonators were dried at 60 °C for 12 h to evaporate the solvent. The collectors with the conductive glue were then evenly coated with the electrode slurry using a drop‐casting procedure with a wet drop mass of ≈230 µg. The resonators were subjected to the same drying procedure as described above. Ultimately, the mass loading of the dry carbon coating did not exceed 50 µg (0.285 mg cm^−2^), ensuring a thin and rigid coating layer necessary for the EQCM application, Figure S4 and Table S3 (Supporting Information) (to consider Saurbrey equation, Equation S1, Supporting Information). The same electrode material was used to cover current collectors in the Swagelok‐type cell (denoted as Swagelok) and volume cell. However, in this case, the active mass load was much higher (≈6.5 mg (5.75 mg cm^−2^)). All the specific capacitance values discussed were related to the active mass of the electrode material.

### Elemental Analysis

The determination of the mass fractions of carbon, hydrogen, nitrogen, and oxygen was carried out with ThermoFisher Scientific FlashSmart (USA) equipment. The amount of oxygen was obtained through direct (separate) elemental analysis. The results are the average values of three separate analyses (Table S2, Supporting Information).

### Electrolytes

The following compounds were used in the study: lithium nitrate (LiNO_3_), lithium sulfate (Li_2_SO_4_), potassium iodide (KI), and solvents: DI water, and deuterium oxide (D_2_O) with purity ≥99,5% (Sigma–Aldrich; USA). Electrolyte solutions (0.1 mol L^−1^ for LiNO_3_, Li_2_SO_4_, and KI) were prepared in distilled water with an electrical conductivity <2 µS cm^−1^ (water purification system; Hydrolab; Poland). Furthermore, a 0.1 mol L^−1^ solution of LiNO_3_ was prepared in D_2_O (with conductivity ca. 20 µS cm^−1^). The conductivity and pH of the given electrolytes are provided in Table S4 (Supporting Information).

### Electrochemical Cells

The electrochemical cell consists of a polyetheretherketone (PEEK) body with a structure adapted to electrochemical measurements with the implementation of EQCM (Figure S5, Supporting Information). The resonator with an AC coating was placed at the bottom of the cell and served as the working electrode (WE). A stainless‐steel foil was used as the counter electrode (CE) with a geometrical area that exceeded that of the WE (20 cm^2^ of CE vs. 0.21 cm^2^ of WE). A saturated calomel electrode (SCE; 0.241 V vs. SHE) was placed close to the resonator and acted as the reference electrode (RE). An excess of electrolyte (400 µL) was injected into the cell, ensuring that there were no trapped air bubbles. In a symmetric Swagelok cell consisting of a poly(tetrafluoroethylene) (PTFE) body, the WE and CE were separated by two GF/A porous membranes (Whatman; USA; d = 12 mm; thickness 260 µm). For this system, SCE and Hg/Hg_2_SO_4_/0.5 mol L^−1^ K_2_SO_4_ were used as reference electrodes. The same reference electrodes were used for the “in‐house made” volume cell (also made out of PTFE). The volume cell provided construction conditions similar to those of the EQCM cell (excess of the electrolyte, oversized CE, and a large distance between WE and RE).

### Electrochemical Investigation

All electrochemical measurements were performed on a multichannel potentiostat/galvanostat VMP3 (BioLogic; France). A QCA922 quartz analyzer (SEIKO EG & G; Japan) was connected to a potentiostat/galvanostat using an analog ±10 V BNC connection (for monitoring the frequency and resistance change). In this way, the QCM analyzer is controlled in the EQCM mode by the potentiostat/galvanostat during electrochemical measurements. The measurements (CV, SPECS controlled by chronoamperometry, and SPEIS) were conducted using EC‐Lab software (BioLogic; France).

The electrochemical stability range was selected during preliminary experiments in a very broad potential range carried out in the EQCM cell (−1.3–1 V vs. SCE; Figure S6, Supporting Information). The scan rate (1 mV s^−1^) was selected based on its sensitivity to all side reactions. The limiting potential values were selected to avoid the presence of water decomposition peaks. Registered preliminary data were not subjected to further interpretation. For LiNO_3_ and Li_2_SO_4_ tests_,_ the chosen potential window was equal to −1 to 0.6 V vs. SCE, and for KI, it was −1 to 0.4 V vs. SCE, which was consistent with other reported data.^[^ ^8^, ^31^, ^35^ ^]^

On the first assembly of the EQCM system, the experiments listed below were performed to determine the PZC:

1.1) 3 cycles of CV at 50 mV s^−1^ from open circuit voltage (OCV) and finished at OCV (system conditioning)
1.2) 1 cycle of CV at 1 mV s^−1^ from OCV and finished at minimum potential (*E_min_*)
1.3) 1 cycle of SPECS from *E_min_* and finished at *E_min_*
1.4) 1 cycle of SPEIS from *E_min_* and finished at *E_min_*

Data from experiment 1.2) were used for further comparison with SPECS and SPEIS data to specify the PZC. For further CV technique discussion, please see Figure S7 and Equation S2 (Supporting Information). SPECS is based on a series of equal‐magnitude potential steps (*△E* = 10 mV; controlled by chronoamperometry) with a rest time of 10 s. In the rest time, the current equilibrium (*I–t* transient) is established for each potential step. The selection of the potential step and the rest time during the SPECS experiment is related to the conductivity of the aqueous electrolyte. For further SPECS technique discussion, please see Equation S4 (Supporting Information). SPEIS begins with *E* = 200 mV in the frequency range from 10 kHz to 1 mHz (sinus amplitude *v_a_* = 5 mV; points per decade *n_d_* = 10; measures per frequency *n_a_* = 1; with drift correction). For further SPEIS technique discussion, please see Figure S8 and Equation S3 (Supporting Information).

The second set of experiments aimed to investigate the EQCM changes, as is listed below.

2.1) 3 cycles of CV at 50 mV s^−1^ from OCV and finished at OCV (system conditioning)
2.2) 20 cycles of CV at 5 mV s^−1^ in the wide potential range (second system)

Only systems with stable performance, where the recorded resistance change was less than ±2% were considered for further mass recalculation (Δ*R* = ±2%). The mass change study using the determined PZC value combines electrochemical data (controlled by a potentiostat, *I*‐*E* curves) and the simultaneously recorded change in the resonator frequency (measured by the EQCM, Δ*f*). The change in resonator frequency could be directly recalculated into the mass change with respect to Equation S1 (Supporting Information).

Faraday's law (Equation 1) was used to recalculate the CV data, allowing a comparison of experimental and theoretical mass changes in the mass:charge ratio plot. In this equation, *ΔQ* is charge exchanged [C], *F* is Faraday's constant [96 485 C mol^−1^], *M* is the molar mass of adsorbed/desorbed ionic species [g mol^−1^], and *z* is the number of exchanged electrons (i.e., the valance number of adsorbed/desorbed ions) [‐]).

(1) $$\Delta m=\frac{\Delta Q\cdot M}{F\cdot z}$$

In the Swagelok cell, the potential range of both electrodes working with 0.1 mol L^−1^ LiNO_3_ was determined in the 2‐electrode system using CV at 1 mV s^−1^ (5 cycles from OCV to 1.6 V; considering voltage range from EQCM study, Figure S6, Supporting Information). The next procedure involved 3 CV cycles at 1 mV s^−1^ in a predetermined potential window (−0.86–0.74 V vs. SCE) in a 3‐electrode system. The experiments were completed using 1 SPECS cycle (controlled by chronoamperometry) with the same conditions as in EQCM. The same testing protocol, with a 1.6 V operating voltage window, was applied to the volume cell.

## Conflict of Interest

The authors declare no conflict of interest.

## Author Contributions

S.S. and P.G. are co‐first authors and contributed equally to the article. S.S. performed methodology, investigation and conceptualized the project, and wrote the original draft. P.G. performed methodology, investigation, and data curation, conceptualized and visualized the project, and wrote the original draft. A.P.‐M. conceptualized, supervised, and visualized the project, performed methodology, and data curation, and wrote the original draft. J.M. acquired funds, supervised the project, and wrote, reviewed & edited the final manuscript. S.W.D. performed validation, supervised the project, and wrote, reviewed & edited the final manuscript. K.F. acquired funds, supervised the project, and wrote, reviewed & edited the final manuscript.

## Supporting information



Supporting Information

## Data Availability

The data that support the findings of this study are available from the corresponding author upon reasonable request.
