# Supplementary material for: Fundamentals and Implication of Point of Zero Charge (PZC) Determination for Activated Carbons in Aqueous Electrolytes
Source: Adv Sci (Weinh). 2024 Nov 13;11(48):2409162. doi: 10.1002/advs.202409162 (PMC11672325; doi:10.1002/advs.202409162)
Supplement: Supplementary file 1 — Supporting Information [file ADVS-11-2409162-s001.docx]

**Supplementary information**

for

**Fundamentals and implication of PZC determination for activated carbons in aqueous electrolytes**

Sylwia Slesinska^1^*, Przemysław Galek^1^*, Jakub Menzel^1^, Scott W. Donne^2^, Krzysztof Fic^1^**, Anetta Płatek-Mielczarek^1,3,4^**

^1^Poznan University of Technology, Institute of Chemistry and Technical Electrochemistry, Berdychowo 4, 60965, Poland

^2^Discipline of Chemistry, University of Newcastle, Callaghan, NSW, 2308 Australia

^3^Laboratory for Multiphase Thermofluidics and Surface Nanoengineering, Department of Mechanical and Process Engineering, ETH Zurich, Sonneggstrasse 3, 8092, Zurich, Switzerland

^4^Unbound Potential GmbH, Bönirainstrasse 14, 8800, Thalwil, Switzerland

*Authors with the equal contribution in article

**Corresponding authors: aplatek@ethz.ch, krzysztof.fic@put.poznan.pl

ORCID:

Slesinska S.: 0000-0002-9242-5561

Galek P.: 0000-0001-9841-9616

Menzel J.: 0000-0002-0431-159X

Donne S.W.: 0000-0001-9389-7870

Płatek-Mielczarek A.: 0000-0001-6231-3908

Fic K.: 0000-0002-5870-7119

**Contents**

EQCM theoretical introduction 3

AC characterization 4

Porous electrode coatings 6

Electrolyte characterization 8

EQCM system 8

Wide potential range screening in EQCM system 9

Discussion 9

CV technique for PZC determination 9

SPEIS technique for PZC determination 11

SPECS technique for PZC determination 13

Minimal specific capacitance variations 14

Influence of the applied potential range on the position of PZC 16

Cell construction for PZC determination 16

Reference influence on the electrochemical operation 20

PZC determination for planar resonator and AC coatings 21

EQCM system verification – comparison to literature data 23

D_2_O as a solvent 24

References: 26

# EQCM theoretical introduction

EQCM consists of a thin piezoelectric quartz crystal placed between two metal electrodes used to apply an alternating electric field across the crystal. This causes a vibrational motion of the crystal at its resonance frequency.^1,2^ The Saurbrey equation (**Eq. S1**) can then be applied to convert the frequency changes (*△f*; Hz) to the mass changes (*△m*; g). In this equation, *A* is surface area of quartz crystal [m^2^], *ρ* is density of quartz (2.648 g cm^-3^), $\text{μ}$ is shear modulus of quartz (2.947 10^11^ g cm^−1^ s^−2^), *△f* is change in frequency [Hz], and *f_0_* is fundamental resonance frequency of the crystal [Hz].

$\text{∆m}\text{ }\text{=}\text{ }\text{-}\text{ }\frac{\text{∆f}\text{ }\text{∙}\text{ }\text{A}\text{ }\text{∙}\text{ }\sqrt{\text{ρ}\text{ }\text{∙}\text{ }\text{μ}}}{\text{2}\text{f}_{\text{o}}^{\text{2}}}$ (**S1**)

At PZC, EDL forms spontaneously because of the natural potential difference between the electrodes and unequilibrated surface charge at the maximum entropy state of the interface. At this point, the highest disorder of the interfacial water is observed.^3,4^

Thus, to quantitatively correlate the mass changes of the adsorbed ions and solvent molecules in EQCM, PZC should be assigned in a correct manner. The determination of adsorbed species is based on a mathematical model that results from Faraday’s law and the mass change/charge relationship. In the case of one-element deposition or dissolution processes on the planar metallic surfaces, the mass change can be easily correlated with ongoing electrochemical processes.

Because PZC corresponds to an electrostatic electrode-electrolyte interaction (EDL formation only), diffusion-limited processes such as redox reactions should be excluded altogether. This is why the popular approach based on the determination of PZC through measured minimum capacitance from CV (related to the semiconducting behaviour of highly porous disordered carbons)^5^ or SPEIS, as demonstrated in many works,^4,6-11^ seems confusing for that reason. Also, when PZC is established, it is not always done in the same system as EQCM measurements.^12^ It can lead to a different starting point in data evaluation, which can further cause an unrationed process description. Such a divergence can also result from cell construction limitations: size, distance between and type of electrodes, volume of electrolyte, and spatial organisation – what will be discussed within this manuscript.

# AC characterization


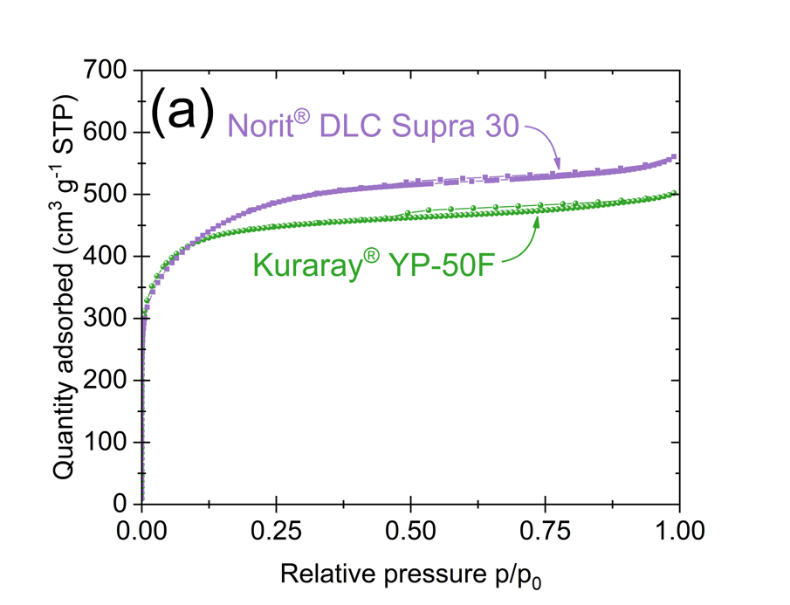

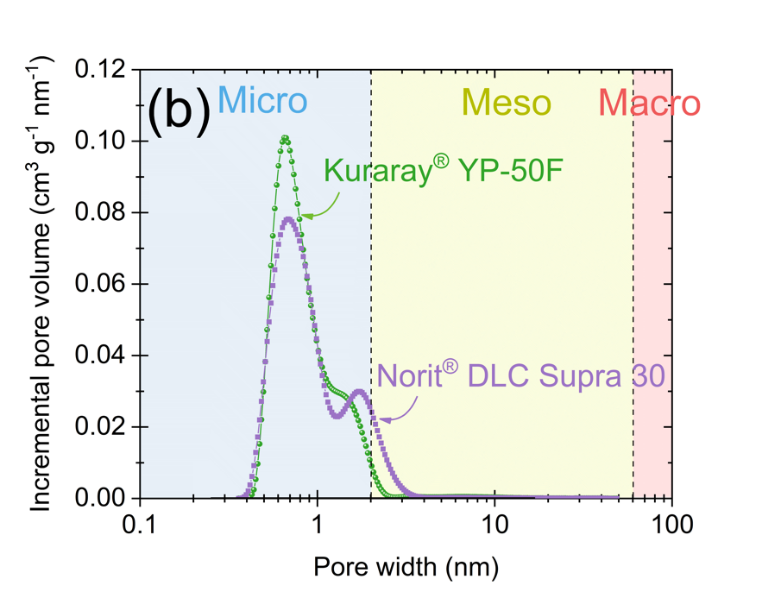


**Fig. S1.** Nitrogen adsorption at 77 K (**a**) isotherms and (**b**) pore size distributions of powder AC YP-50F and DLC30.

The textural properties of the YP-50F and DLC30 ACs are presented in **Fig. S1 and Tab. S1**. Nitrogen adsorption/desorption isotherm was recorded at 77 K (ASAP 2460; Micrometrics^®^, USA) to evaluate the porous texture. Prior to analysis, ACs were purged under helium flow for 12 h at 350˚C and then placed under high vacuum for 5 h. The Brunauer–Emmett–Teller (SSA) equation was used to calculate the surface area at relative pressure range (0.01 – 0.05). Two-Dimensional Non-Local Density Functional Theory (2D-NLDFT) was applied to determine micro and mesopore volume values. The average pore diameter was obtained from the maximum peak value.

**Tab. S1**. Textural properties of AC YP-50F and DLC30 from nitrogen sorption/desorption tests at 77 K

|  | **SSA, m^2^ g^-1^** | **V_micro_, cm^3^ g^-1^** | **V_meso_, cm^3^ g^-1^** |
| --- | --- | --- | --- |
| YP-50F | 1702 | 0.64 | 0.08 |
| DLC30 | 1780 | 0.66 | 0.25 |


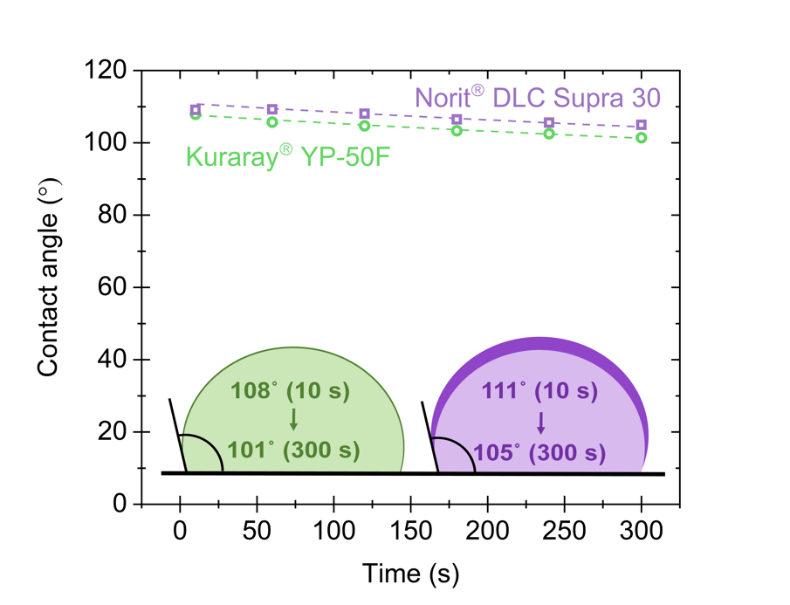


**Fig. S2.** Comparison of changes in dynamic contact angle during 300 s for H_2_O on YP-50F and DLC30 based electrodes.

The contact angle measurements were performed with a computer-controlled goniometer (Dataphysics^®^ OCA). The carbon samples in the form of coatings on steel foil (diameter 2 x 1 cm) were placed horizontally in front of the contact angle camera. The 2 μL volume drops of H_2_O were injected with speed injection 0.5 μL s^-1^ on the surface of the electrode material.

**Tab. S2.** Elemental composition tested by elemental analysis for ACs: YP-50F and DLC30.

| **Activated carbon** | **C (%)** | **H (%)** | **N (%)** | **S (%)** | **O (%)** | **Total (%)** |
| --- | --- | --- | --- | --- | --- | --- |
| Kuraray^®^ YP-50F | 96.1 | 0.8 | 0 | 0 | 2.1 | 98 |
| Norit^®^ DLC Supra 30 | 95.2 | 0.5 | 0 | 0 | 2.2 | 98 |

Based on elemental analysis (EA) data, one can see that YP-50F and DLC30 carbons are almost identical in terms of elemental composition as presented in **Tab. S2.** They are equally oxidized in bulk (~2%). To test the wettability of both carbons, contact angle measurements with H_2_O were performed, as described in **Fig. S2.** The results confirm that both carbons display hydrophobic characteristics with almost identical wettability *∆θ* = ~3°.That would explain why: DLC30 and YP-50F exhibit a similar PZC region in contact with the same aqueous liquid electrolyte; however, the value of specific capacitance can be correlated with their textural properties, and not with their surface chemistry. (**Fig. S1**).

## Porous electrode coatings

The resonator preparation procedure is presented in **Fig. S3**.


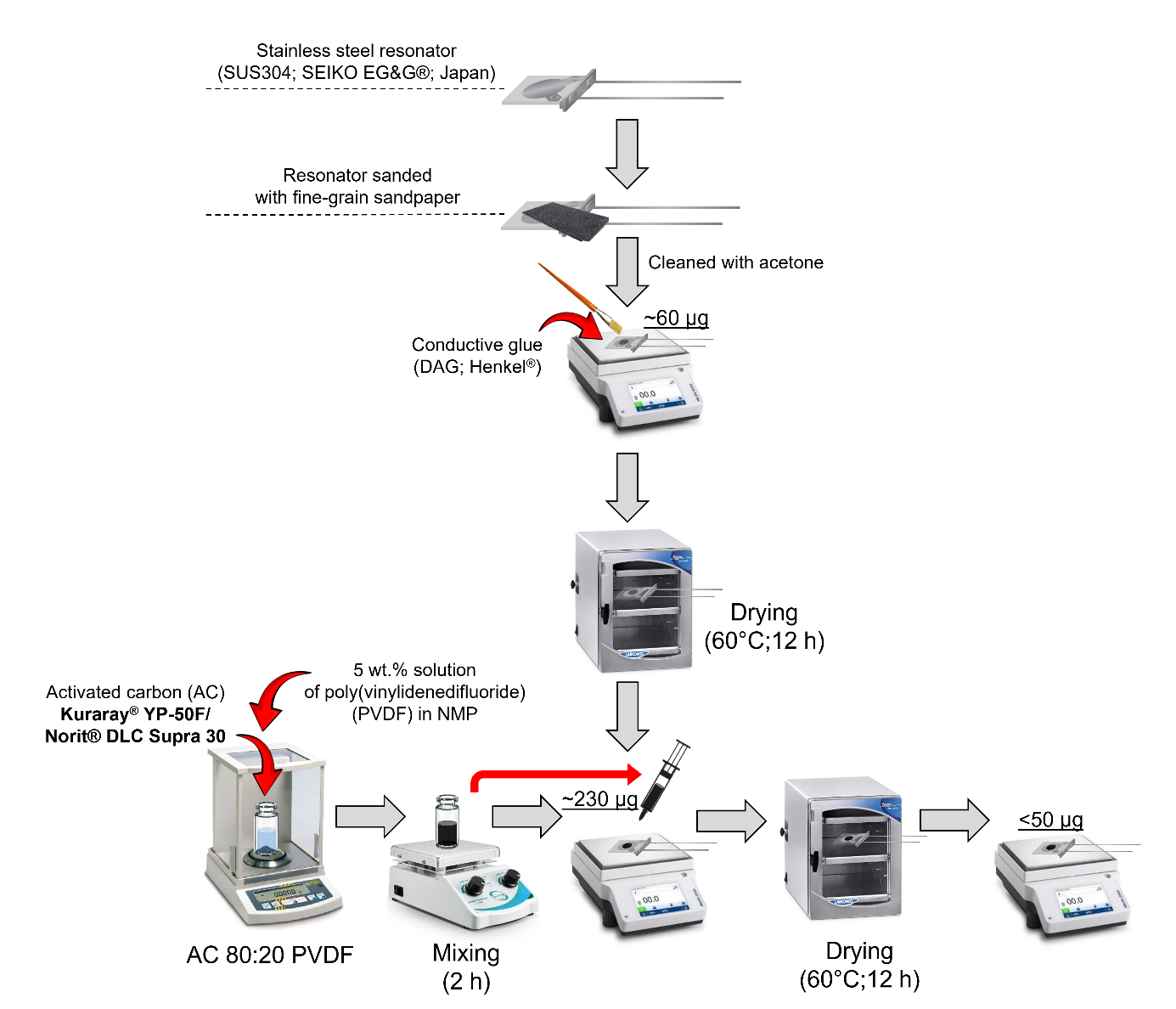


**Fig. S3.** The scheme of the coating preparation on the resonator surface.

SEM micrographs of prepared resonators are presented in **Fig. S4**.

| 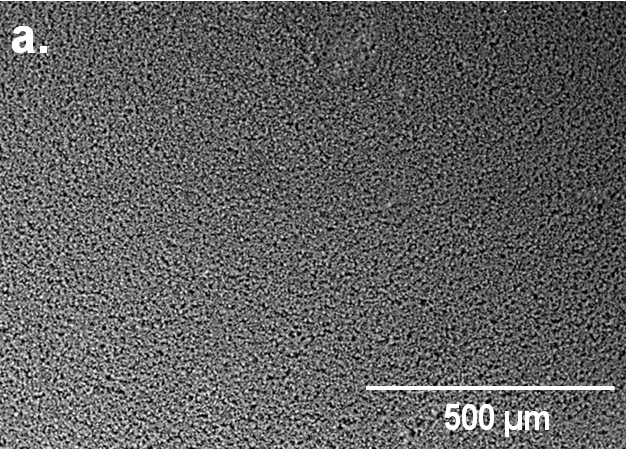 | 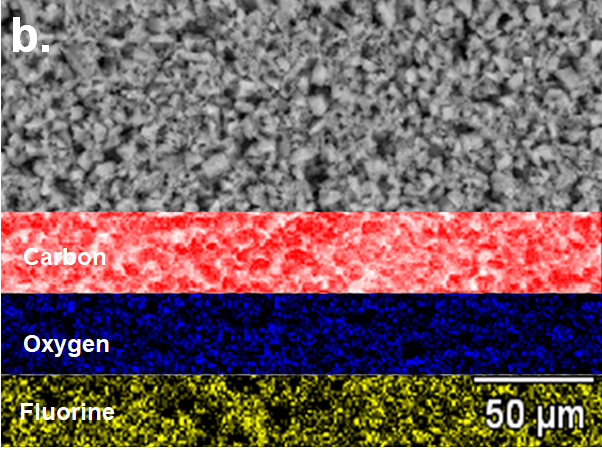 |
| --- | --- |
| 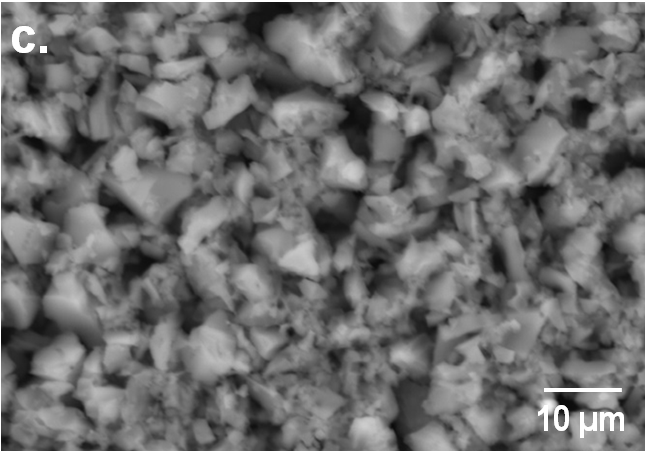 | 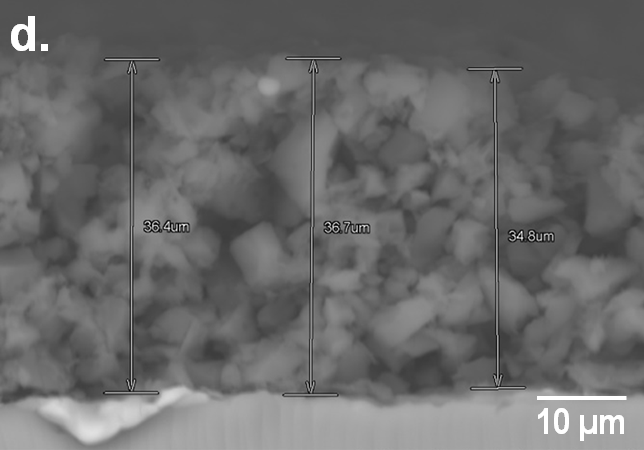 |

**Fig. S4.** SEM micrographs from top view a)-c) and cross-sectional d) for YP-50F coated resonator; b) EDX mapping of C, O, F chemical elements.

Resonators are homogenous and uniform (thickness ca ~35 µm). Carbon particles retain their shape and size comparing to the pristine one. Composition of YP-50F coating on resistor is presented in **Tab. S3** below:

**Tab. S3**. Weight % Elemental composition of the coating from the EDX analysis

| **C (%)** | **O (%)** | **F (%)** | **Total (%)** |
| --- | --- | --- | --- |
| 81.9 | 6.5 | 11.6 | 100 |

# Electrolyte characterization

**Tab. S4.** Conductivity and pH of tested electrolytes with a concentration of 0.1 mol L^‑1^.

| **Solvent** | **Salt** | **Conductivity (mS cm^-1^)** | **pH** |
| --- | --- | --- | --- |
| H_2_O | LiNO_3_ | 9.2 | 6.8 |
|  | Li_2_SO_4_ | 14.6 | 7.4 |
|  | KI | 13.2 | 7.8 |
| D_2_O | LiNO_3_ | 5.8 | 8.8 |

All studied electrolytes can be classified as aqueous neutral solutions (pH in the range of 6-8). This implies, that balance between alkaline and acidic ionic species is preserved. Low concentration solutions were selected based on EQCM sensitivity – therefore, conductivity values of such solutions are rather low. pH and conductivity were measured using Seven InLab Mettler Toledo^®^ (USA) with appropriate sensor for aqueous solutions.

# EQCM system


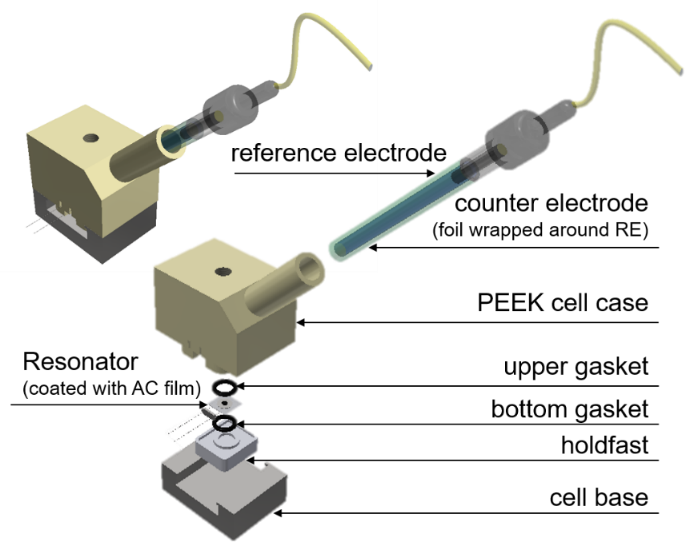


**Fig. S5.** EQCM cell scheme.

# Wide potential range screening in EQCM system


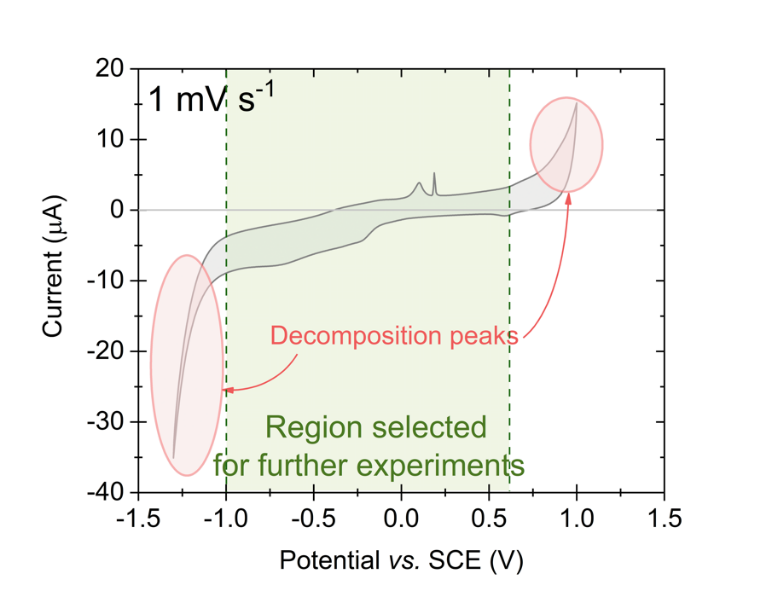


**Fig. S6.** Cyclic voltammogram for YP-50F and 0.1 mol L^-1^ LiNO_3_ in EQCM system at 1 mV s^-1^.

# Discussion

## CV technique for PZC determination

The CV technique allows to calculate the capacitance per electrode *C_CV(v)_* (according to the equation **Eq. S2**, where: *I* – current [A], *v* – scan rate [mV s^-1^]).

$\text{C}_{\text{CV}\text{ }}\text{=}\text{ }\frac{\text{2}\text{ }\text{∙}\text{ }\text{I}}{\text{v}}$ (**S2**)

For aqueous based electrolytes, especially with redox-active species present, one must thus consider applicable scan rate. **Fig. S7** presents cyclic voltammograms for the same system recorded using different scan rates, i.e., 1, 5, and 50 mV s^-1^.


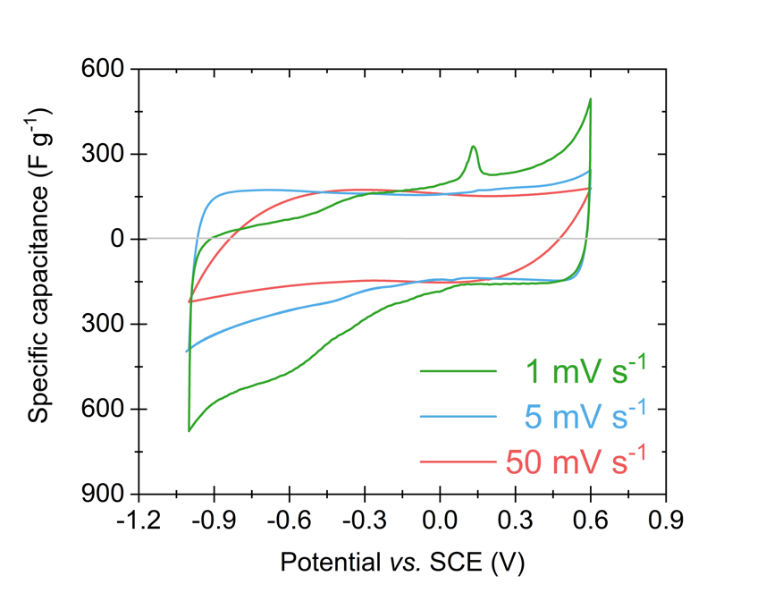


**Fig. S7.** Specific capacitance *vs*. potential based on cyclic voltammetry for 0.1 mol L^-1^ LiNO_3_ and YP-50F at 1, 5, and 50 mV s^-1^ in the EQCM cell.

The CV profile recorded at slow scan rate (1 mV s^-1^) represented the most detailed characterisation of the charging/discharging profile. The moderate scan rate, such as 5 mV s^-1^, effectively depicts pure EDL behaviour – covering all current increase points, where certain redox/side reactions can occur. This is a satisfactory scan rate to characterise the performance of a device, considering that EC is supposed to operate at high power/current loads. The fast scan rate, like 50 mV s^-1^, is, however, too fast to allow any ion flux to easily form EDL at the highly developed electrode surface area (**Fig. S1**) in aqueous solutions with moderate conductivity values (i.e., low concentrations). Thus, we begin to observe resistance components and delayed charge transfer at the electrode/electrolyte interface. One cannot withdraw any information on the charge storage mechanism while using fast scan rates. However, such a change in voltage/potential brings about other useful information – not considered for PZC determination. Therefore, for fundamental studies of aqueous electrolytes that focus on a molecular-level interaction between ionic species and electrode surface, slow scan rates (like 1 mV s^-1^) and will be used in this study.

## SPEIS technique for PZC determination

The SPEIS technique allows to calculate the capacitance *C_SPEIS(f)_* (according to the equation **Eq.** **S2,** where: *f* – frequency [Hz], *-Im(Z)* – imaginary *Z* value [ohm]) (example given in **Fig. S8a**).

$\text{C}_{\text{SPEIS(f)}}\text{ }\text{=}\text{ }\frac{\text{1}}{\text{π}\text{ }\text{∙}\text{ }\text{f}\text{ }\text{∙}\text{ }\text{-Im}\left( \text{Z} \right)}$ (**S3**)

According to the given equation, *C_SPEIS(f)_* is variable and strongly depends on the chosen frequency value. Therefore, in addition to Nyquist plot (**Fig. S8a**), specific capacitance *vs.* frequency is generally reported for electrochemical capacitors (**Fig. S8b**).^13,14^


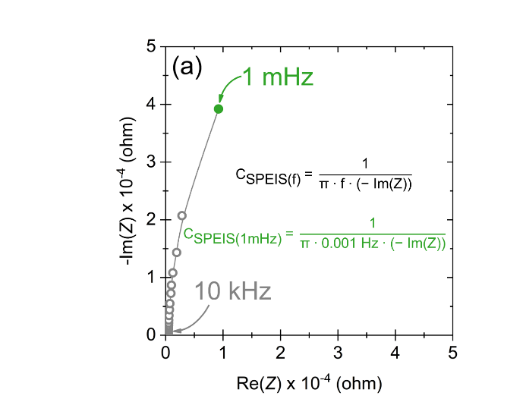

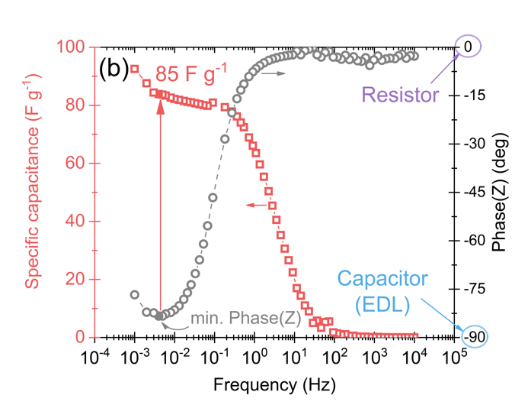

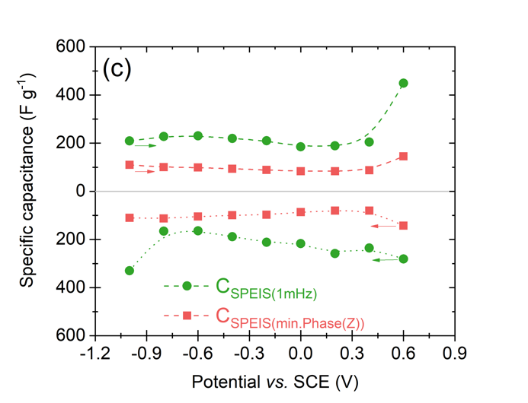


**Fig. S8.** Impedance study at 0 V *vs.* SCE for 0.1 mol L^-1^ LiNO_3_ and YP-50F: (**a**) Nyquist and (**b**) Bode plot (**c**) Comparison of capacitance calculated for 1 mHz (green circles) and determined based on minimum *Phase(Z)* value (red squares).

Therefore, the selection of an adequate frequency for the specific capacitance calculation is crucial to obtain a representative curve that allows the determination of PZC. It should be remembered that choosing a specific frequency range does not necessarily result in an inflection (which corresponds to PZC) on the *C_SPEIS(f)_* curve on *C_SPEIS(f)_* *vs.* potential plot (see *C_SPEIS(1mHz)_* presented in **Fig. S8c**). Thus, with this technique, it is possible (with significant error) to determine the PZC – but only for some experimental conditions. For example, finishing the experiment at a high frequency will only correspond to the data recording related to the system resistance (*R*) (resistive response with *Phase(Z)* = 0°), which would not allow for the full development of the EDL (capacitor response with *Phase(Z)* = ~ -90°) (**Fig. S8b**). If, on the other hand, *C_SPEIS(f)_* is calculated on the basis of the low frequency, then apart from pure *C_EDL_, the* capacitance related to side reactions is registered. It particularly concerns measurements in the extreme potentials, where the response resulting from electrolyte decomposition will be additionally registered. Faradaic charging becomes more predominant at low frequencies, whereas one must remember that the operating frequency cannot be excessively low for capacitive charging. The optimal frequency for capacitive charging is strongly dependent on electrode geometry and electrolyte conductivity.^15^ Changing the direction of polarisation (*E_max_* ⟶ *E_min_*) results in different *C_SPEIS (1mHz)_* than those calculated under *E_min_* ⟶ *E_max_* polarisation (**Fig. S8c**). The *C_EDL_* response should be symmetric about the Y axis for capacitive systems. Surprisingly, upward capacitance, differences in values, and divergence in the capacitance trend in both polarisation directions are observed, probably because of irreversible oxidation processes that occur at the electrode/electrolyte interface at relatively high potentials (close to the oxygen evolution reaction – OER). Nevertheless, there is also another possibility to indicate PZC from SPEIS more accurately. *C_EDL_* can be read from the Bode plot (*C_SPEIS(min.Phase(Z))_*; **Fig. S8b**). As already mentioned, *Phase(Z)* = -90° and 0° correspond to an ideal capacitor and resistor response, respectively.^16,17^ Under real conditions, the pure *C_EDL_* can be read from the closest point to *Phase(Z)* = -90° (inflection point). By this method, it is possible to accurately determine *C_EDL_* recorded for the minimum *Phase(Z)* value, but not without any problems. Firstly, it is necessary to prepare preliminary experiments (a trial setup), for which the frequency range will be selected with an inflection of *Phase(Z)* close to - 90°. A standard frequency range of 100 or 10 kHz to 1 mHz may not result in the Bode plot bending near *phase (Z)* = -90°. Furthermore, one needs to increase the number of points collected (by decreasing *△E*) in the tested potential range to obtain representative results with a clear minimum *C_EDL_* on the *C_EDL_* *vs.* potential plot. Furthermore, the greater the accuracy of data collection (at a given potential step), the longer the total time of SPEIS experiment (for each potential step it can last from a few minutes up to a few hours). For example, for the aqueous based systems presented in this study, single impedance (at given potential) was recorded for 34 min. Such a long holding time of a given potential step (especially in the extreme potentials) in the EQCM system, results in the electrolyte decomposition and its evaporation. At the same time, *△E* = 0.2 V chosen here, for each PEIS measurement, leads to an incomplete spectrum in the investigated potential range; however, this is a compromise between the overall experimental time and the number of collected points. Another disadvantage of the step potential shift is the abrupt occurrence of potentially occurring reactions (e.g., redox) and the lack of time for the ion reorganisation participating in EDL formation. The gradual change of the potential has a better effect on the stabilised reorganisation of the charge on the surface of the electrode material. Such charging procedure differs from those in commercial systems, where the charging process is constant. However, apart from those disadvantages, it seems that the PZC value is more realistic when considering the minimum capacitance based on the minimum of Phase(Z) instead of the lowest frequency value. More observations have been discussed for *C_SPEIS(min.Phase(Z))_* and *C_SPEIS(1mHz)_* for different types of electrolytes in the subsequent part of the article.

## SPECS technique for PZC determination

SPECS technique allows to separate the total system capacitance (*C_T_*) calculated at a given potential step into individual capacitances corresponding to: EDL capacitance component *C_EDL_* *=* *C_P_* *+* *C_G_* (pure capacitor response) part of the porous (*C_P_*) and geometrical (*C_G_*) area of the electrode. Moreover, it is possible to separate capacitance resulting from ion diffusion (*C_D_*) and residual capacitance (*C_R_*) mainly related to redox and side reactions (e.g., electrolyte decomposition). In summary, *C_T_* can be described as *C_T_* = *C_P_* *+* *C_G_* + *C_D_* + *C_R_*. By dividing the area of individual current curves *I_P_, I_G_*, *I_D_*, *I_R_* [*⟆A_I_*; A s] registered at each potential step by *△E* and the mass of the electrode active material, it is possible to obtain individual values for the specific capacitance.^18^ A detailed description of this technique can be found in the article presented by M. Dupont and S. Donne.

The specific capacitance of the individual components (C_T_, C_P_, C_G_, C_D_, C_R_) obtained by the SPECS technique was calculated according to equation below.

$\text{C=}\frac{\text{A}}{\text{M∙∆E}}$ (**S4**)

where: C – specific capacitance of given component [F g^-1^]

A – surface area under given calculated current curve [A s]

M – mass of active mass [g]

∆E – potential step (0.01 V) [V]

## Minimal specific capacitance variations

**Tab. S5** PZC region for Li_2_SO_4_ depending on the concentration and minimal specific capacitance variations.

|  |  | **E_min_ *vs.* SCE, V** | **E_max_ *vs.* SCE, V** | **ΔE, mV** |
| --- | --- | --- | --- | --- |
| ±1% | 0.1 mol L^-1^ | -0.02 | +0.01 | 30 |
|  | 1 mol L^-1^ | +0.05 | +0.23 | 180 |
| ±2% | 0.1 mol L^-1^ | -0.04 | +0.05 | 90 |
|  | 1 mol L^-1^ | +0.01 | +0.32 | 310 |

In **Tab. S5** two different concentrations are compared 0.1 and 1 mol L^-1^ for Li_2_SO_4_ and in addition, two specific capacitance ranges for minimal value, ±1 or ±2%. One can observe that the higher the specific capacitance deviations, the wider the RZC. We opt that ±1% is not a sufficient range to consider for porous AC electrodes, especially for samples with quinone/hydroquinone redox pair activity. What is important to note is that the higher the electrolyte concentration, the wider the RZC. It is related to the number of charges accumulated at the electrode/electrolyte interface and in effect, wider potential zone of ion permselectivity failure of porous electrode. We assume that more porous electrode material could lead to the extension of RZC for specific concentration of aqueous solutions. Higher aqueous solution concentration causes slight shift of RZC towards more positive potential values, what is caused by a higher quinone/hydroquinone redox peak activity at ca. 0.15 V *vs*. SCE in Li_2_SO_4_.

Considering all above-mentioned observations, we do postulate that capacitance variation ±2%, which corresponds to ~1 F g^-1^ difference, is a reasonable potential range for a porous electrode material. Knowing experimental conditions and mathematical calculations, bigger accuracy of specific capacitance values is meaningless. Surface functionality redox reactions are very sensitive to aqueous environment, thus, selection of PZC instead of RZC can be fraught with more error and difficulty, which proves non-universality of this property for porous carbon materials. Additionally, porous electrode materials owing to their developed textural properties should be combined with diluted aqueous solutions to preserve required experimental conditions for Saurbrey equation application (ΔR < 2%) which can be observed in the literature data presented in **Fig. 3** (diluted solutions are mostly discussed).


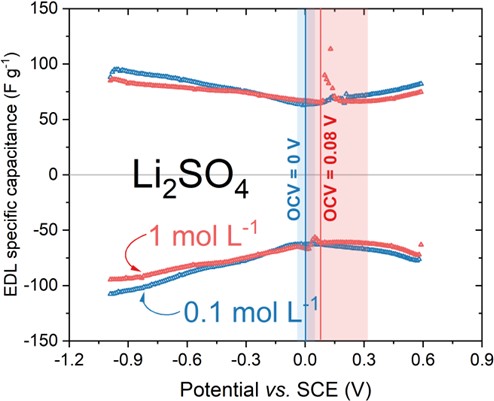


**Fig. S9.** PZC determination from specific capacitance vs. potential for Li_2_SO_4_ in two concentrations: 0.1 and 1 mol L^-1^.

**Tab. S6.** Ionic radius and RZC for anions in electrolytes used in this research and RZC (taking into account minimum capacitance value ±2%).

| **Electrolyte** | **Anion** | **Ionic radius^[12, 13]^** | **RZC** |
| --- | --- | --- | --- |
| 0.1 M Li_2_SO_4_ | SO_4_^2-^ | 0.242 nm | 90 mV |
| 1 M Li_2_SO_4_ | SO_4_^2-^ | 0.242 nm | 310 mV |

## Influence of the applied potential range on the position of PZC


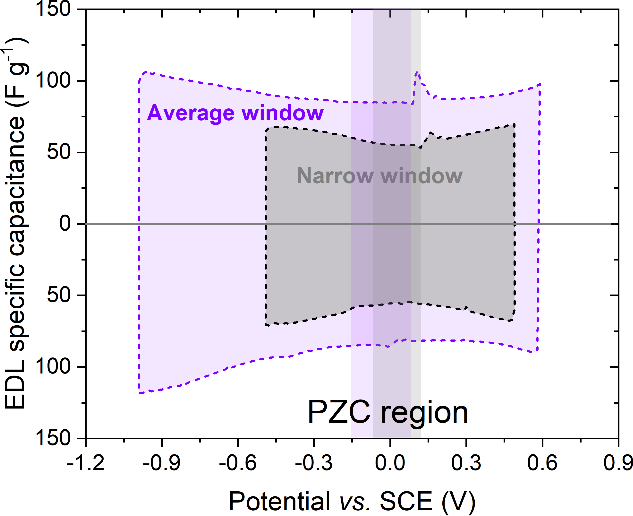


**Fig S10.** Influence of the applied potential range on the position of PZC in the 0.1 mol L^-1^ LiNO_3_ electrolyte in the EQCM system.

**Tab. S7.** RZC for LiNO_3_ studied in a narrow (*ΔE* = 1 V) and a wide (*ΔE* = 1.6 V) potential window.

|  | **E_min_ *vs.* SCE, V** | **E_max_ *vs.* SCE, V** | **ΔE, mV** |
| --- | --- | --- | --- |
| ΔE = 1.0 V | -0.07 | +0.12 | 190 |
| ΔE = 1.6 V | -0.15 | +0.09 | 240 |

## Cell construction for PZC determination

It is also vital to highlight the influence of the cell itself on the determination of PZC. We have demonstrated that if PZC is obtained in any other setup, i.e., such as Swagelok cell or volume cell, it will differ greatly from PZC in the EQCM cell (**Fig. S11**).


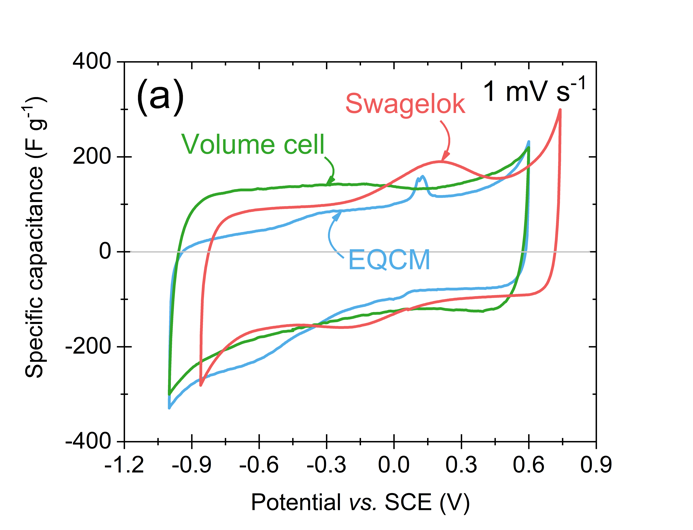

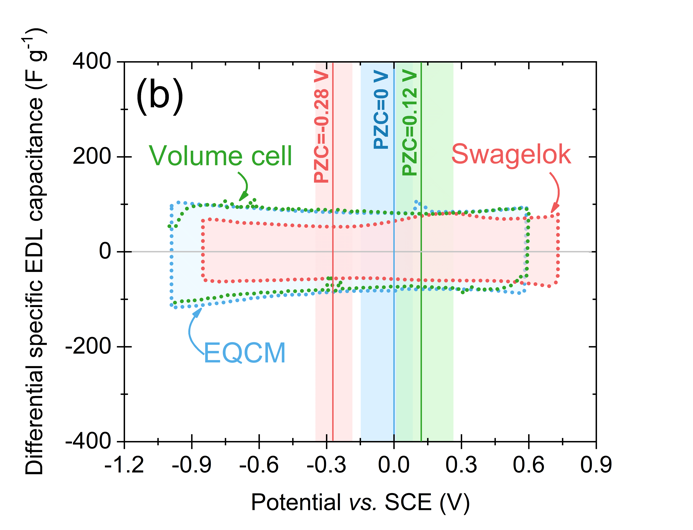


**Fig. S11.** Comparison for Swagelok (red line), EQCM (blue line), and volume cell (green line) (0.1 mol L^-1^ LiNO_3_ electrolyte and YP-50F) of (**a**) specific capacitance *vs*. potential – CV experiment at 1 mV s^-1^ and (**b**) PZC and PZC region from SPECS experiment.

Electrochemical stability for the EQCM and the Swagelok cell differs by ca. 200 mV. This potential shift can be minimised using a similar component ratio as in the EQCM cell represented in the volume cell (WE to CE geometrical surface ratio, excess of electrolyte, CE electrode type). **Fig. S11a** presents the specific capacitance calculated from the cyclic voltammetry data for a scan rate of 1 mV s^-1^ in predetermined potential windows in the given systems. Measurements were made by repolarisation of one electrode in the wide potential range. CV plots for volume cell and EQCM overlap their potential range; however, the EQCM curve represents the highest extent of details (redox contribution). **Fig. S11b** presents the differential specific EDL capacitance (*C_SPECS(EDL)_* = *C_P_* + *C_G_*) calculated based on the SPECS technique and is presented in the same graphical representation as in **Fig. S11a**. The Swagelok and EQCM/volume systems are incomparable because of the mutual shift of the electrochemical stability range, and consequently, the PZC. The ohmic drop associated with the varied distance between RE and WE was initially taken as the cause of the potential shift in Swagelok (**Fig. S12**). However, this does not seem to be an issue.


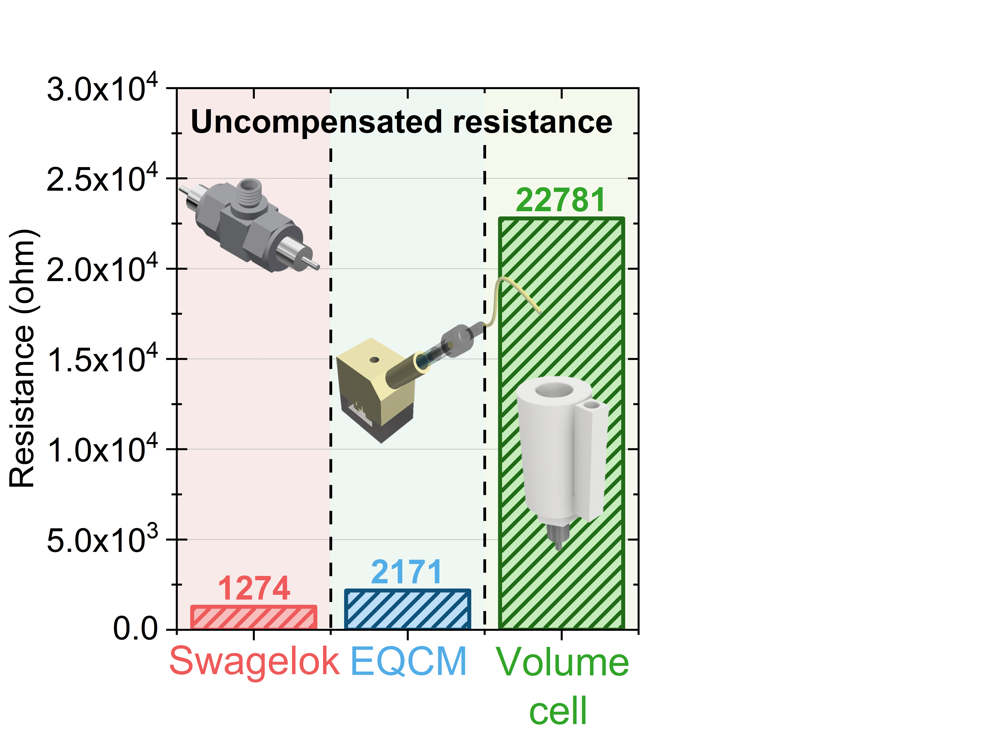


**Fig. S12.** Comparison of uncompensated (*R_u_*) resistance between working and reference electrode for Swagelok, EQCM, and volume cell.

Measurement of uncompensated (*R_u_*) resistances was conducted in 2-electrode configuration (in Swagelok, EQCM, and ‘in-house made’ volume cell) between working and reference electrode using current interrupt (CI) technique. Applied current density was 0.1 A g^-1^ with 80% compensation on *R_u_*, where average values were determined on three cycles. R*_u_* has values: 1274 ohm – Swagelok, 2171 ohm - EQCM and 22781 ohm – volume cell. It shows that the less confine system (large WE and CE distance, excess of electrolyte etc.) leads to higher uncompensated resistance value related with system design.

Although the volume cell has a much higher (several times) uncompensated resistance (*R_u_*) value than the EQCM cell, the operating potential range of both systems is identical. For EQCM and Swagelok, the difference between *R_u-EQCM_* and *R_u-Swagelok_* is less than two times, yet there is a big difference in the electrochemical stability of both systems. It is well known that the potential range is dependent on the pH of the electrolyte. Therefore, the possible effect of pH changes resulting from migration of Cl^-^ ions from SCE to Swagelok was considered and verified. However, this possibility was debunked (**Fig. S13**). In tight systems (without excess of electrolyte), such as Swagelok, the pH can change directly after immersion of the electrode in the electrolyte (without polarisation). This can acidify the medium at the vicinity of the electrodes and is thus the only explanation for the observed shift. For systems with high CE/WE ratio and an excess of the electrolyte such as EQCM and volume cell, this phenomenon is limited. The pH adjustment in such systems is much more efficient because of the facilitated diffusion.

The difference in the value of PZC between Swagelok and other systems (EQCM and volume cell) with 0.1 mol L^-1^ LiNO_3_ and YP-50F is significant (*∆E* = 0.28 V) and can lead to misinterpretation of the data. The potential range for the EQCM (blue line) and volume cell (green line) is identical, which stems from a similar design in both systems (large electrolyte excess and asymmetry between WE/RE). In both cells, the PZC is shifted towards *E_max_* and divided the entire potential window (1.6 V) into given potential ranges *∆E(+)* = 0.6 V and *∆E(-)* = 1 V in EQCM and *∆E(+)* = 0.48 V and *∆E(-)* = 1.12 V in volume cell. In the Swagelok cell, PZC is shifted towards *E_min_* and the individual ranges are *∆E(+)* = 1.02 V, and *∆E(-)* = 0.58 V. Therefore, one can see that even for the capacitive symmetric system (Swagelok cell), the potentials are not divided equally for positive (WE) and negative (CE) electrodes. The construction of the system (size, volume, pressing, etc.) influences the operating potentials of the electrodes. The ohmic drop is a value that can explain this difference. The Ohmic drop is the amount of potential that is lost on the way from the reference electrode to the working electrode and results from the Ohmic resistance between the working electrode and the reference electrode – denoted as R*_u_*. The distance between the electrodes and the conductivity of the solution can thus largely influence its value.

In addition, this non-uniform potential difference between two symmetrical electrodes informs about varied dominant processes during EC charging. Especially on the microscale at the electrode/electrolyte interface, where the equilibrium state cannot be taken for granted for the same system composition (YP-50F + 0.1 mol L^-1^ LiNO_3_) but varies accordingly with the system construction and size. Similarly to the EQCM system, redox in Swagelok induces an increase in the EDL capacitance but in a much wider range of potential. What was noted was that the redox response is not visible in a volume system at all as the probability of the gaseous side products to evolve is higher than their interaction within confined electrode porosity.

## Reference influence on the electrochemical operation


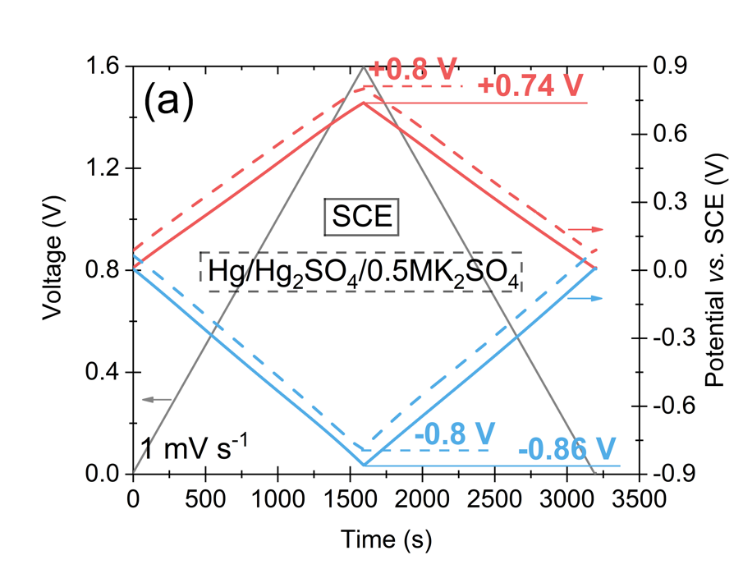

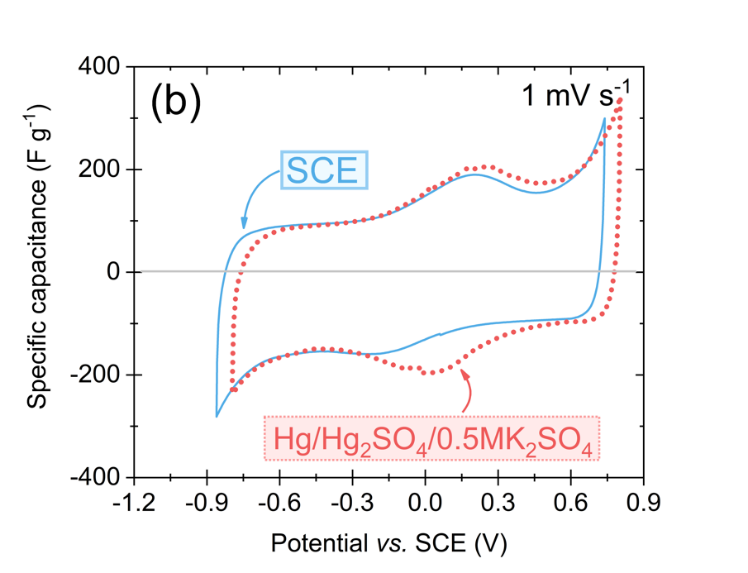


**Fig. S13.** (**a**) Difference in potential shift (for 1.6 V) in 3-electrod and (**b**) 2-electrode configuration using SCE and Hg/Hg_2_SO_4_/0.5 mol L^-1^ K_2_SO_4_ as a RE in 0.1 mol L^-1^ LiNO_3_-based Swagelok system with YP-50F.

Comparison in **Fig. S13a** presents the shift in the potential of positive (red lines) and negative (blue lines) electrode for the Swagelok system with 0.1 mol L^-1^ LiNO_3_, when SCE (solid line) or Hg/Hg_2_SO_4_/0.5 mol L^-1^ K_2_SO_4_ (dashed line) was used as the RE. The potential range was determined using CV with a scan rate 1 mV s^-1^ to 1.6 V (black line). **Fig. S13b** shows specific capacitance (calculated based on CV) for previously mentioned systems and previously determined potential ranges (with the repolarisation of one electrode).

The suspicion of a potential shift in the Swagelok relative to potentials of other systems (EQCM and volume cell) was the possible migration of Cl^-^ ions (as a result of a concentration gradient) from RE into the tested system. This migration of ions could induce a change in the electrolyte pH, and consequently, would lead to a change in the potential range of both electrodes. To eliminate this possibility, the SCE electrode was replaced with Hg/Hg_2_SO_4_/0.5 mol L^-1^ K_2_SO_4_. Salt bridge was used to avoid possible ion migration between the reference electrode and the electrolyte bulk.

The change of RE does not shift potential range towards negative values (comparable to EQCM and volume cell). It can be concluded that the type of RE does not affect such a significant shift of the potential observed in **Fig. S13** in Swagelok in comparison to the potential range of the rest systems. The use of Hg/Hg_2_SO_4_/0.5 mol L^-1^ K_2_SO_4_ shifts the potential even more towards positive values. The difference between the systems (with RE Hg/Hg_2_SO_4_/0.5 mol L^-1^ K_2_SO_4_ *vs*. SCE) is 60 mV. In this case, the potential shift in Swagelok in comparison to EQCM or volume cell results from the design of the cell, not from the pH changes caused from migration of ions from RE (even though Swagelok contains the lowest ratio of electrolyte volume to electrode mass). The close contact of both electrodes, their similar size and mass, and the much smaller volume of electrolyte than in EQCM/volume cell affect the susceptibility of the Swagelok to local pH changes, thus potential shift.

## PZC determination for planar resonator and AC coatings

Another interesting aspect seems to be the determination of the PZC for the EQCM system with a resonator not covered with the carbon material (**Fig. S14**). It is challenging to determine PZC for D_2_O, H_2_O and 0.1 mol L^-1^ LiNO_3_ based system with a planar resonator. For D_2_O, a very wide range (~0.8 V) of minimum capacitance can be observed; for which it is impossible to determine one PZC value. However, for systems with H_2_O and 0.1 mol L^-1^ LiNO_3_, two local minima of the capacitance were registered. Most likely, a minimum close to 0 V *vs.* SCE is a region of the PZC, but it cannot be stated with certainty. These tests show that PZC is a unique property of the electrode material with a developed texture and a broad surface chemistry in contact with the liquid electrolyte.


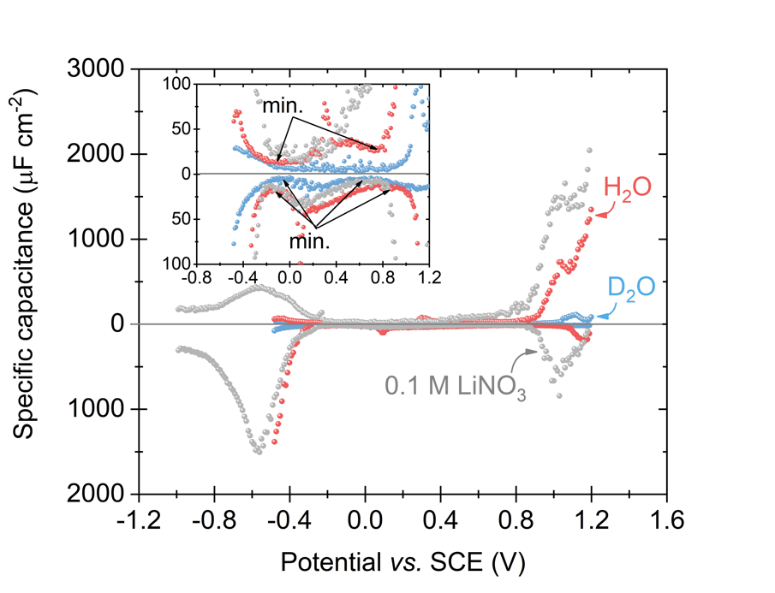


**Fig. S14.** Specific capacitance *vs*. potential for EQCM system with steel resonator (planar) and D_2_O, H_2_O, and 0.1 mol L^-1^ LiNO_3_ as electrolyte.

In the second part of the study, the influence of AC used as electrode material was compared in the PZC region (in the EQCM system with D_2_O as electrolyte) was compared (**Fig. S14**). The location and width of the PZC region (0.02 – 0.9 V *vs.* SCE) are identical, despite the different structural and textural characteristics of both materials. However, there is a difference in capacitance obtained with the SPECS technique; DLC30 based system has higher capacitance compared to YP-50F (**Fig. S15**). Interestingly, the biggest difference in capacitance is observed in the PZC region, while it decreases as extreme potentials are reached. This behaviour is due to the difference in the availability of the porous structure of both materials for any charges coming from the electrolyte. In the PZC region, the driving force (potential value) for ion adsorption is low. The surface, which is readily available for the electrolyte (including mesopores), is mostly charged with ions. The amount of charge adsorbed by DCL30 is greater due to the wider pore distribution range than YP-50F (**Fig. S1b**). In the *E_max_* and *E_min_* regions, the driving force of ion adsorption is large enough to draw the ions into the microporosity structure. The capacitance is equal in these regions for both materials. This proves that the same amount of charge is adsorbed in the microporous structure for both tested materials.


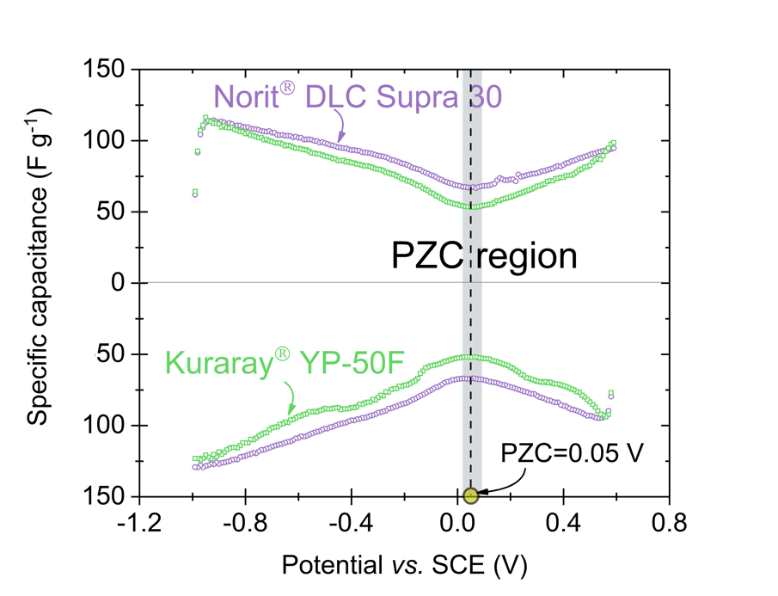


**Fig. S15.** Comparison of PZC region for EQCM system with YP-50F and DLC30 as electrode material (with D_2_O as electrolyte)_._

# EQCM system verification – comparison to literature data

First, in order to verify the operation of the EQCM system, redox electrolytes have been tested, namely: 0.1 mol L^-1^ KI and 0.1 mol L^-1^ RbI [8] – presented in **Fig. S16**. Cyclic voltammograms present typical iodide/iodine redox activity in the positive potential values. The system with KI electrolyte was tested in a narrower voltage window to resemble experimental conditions as for other capacitive electrolytic solutions tested (LiNO_3_ and Li_2_SO_4_). 0.1 mol L^-1^ RbI is not a main electrolyte studied here, it was used for comparison and validation purpose, i.e., feasibility test to the reported literature data. [8] The mass change calculated for these two redox-based systems shows similar hysteresis loops in the range of the redox activity potential. Hysteresis loop, especially while ending at the higher mass change, informs about species trapped in the electrode pores. As gas formation in the I_2_ form is assumed for such a concentration (0.1 mol L^-1^ MI, M = K^+^, Rb^+^), these small gas bubbles can be trapped in the narrow pores and the number of active species at the electrode/electrolyte interface will decrease with time. Moreover, the iodate-based precipitation can be observed over a limited lifetime of iodide-based EC.^19^ The curves presented (specific capacitance and *m* as a function of potential) show that electrode coatings prepared on EQCM quartz crystal resonators work in a stable manner and can be further used for PZC discussion (*ΔR_RbI_* = *ΔR_KI_* = ±1%).


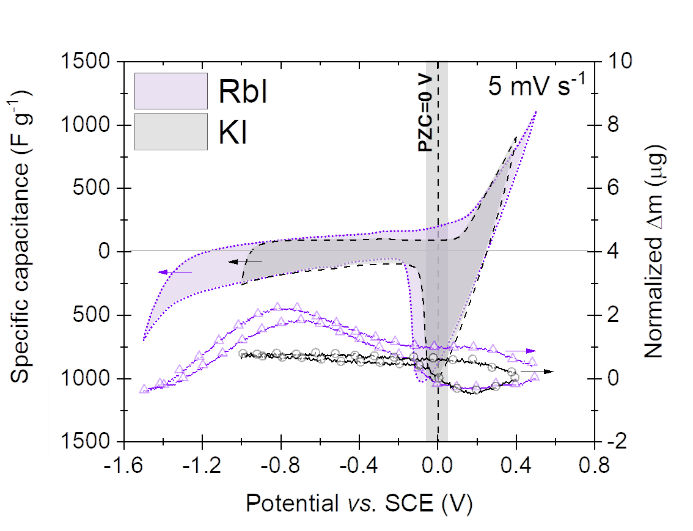


**Fig. S16.** Cyclic voltammetry 5 mV s^-1^ with normalized mass change for EQCM system with 0.1 mol L^-1^ electrolyte: RbI (violet) and KI (black) and YP-50F.

**Tab. S8.** Molar mass of ionic species and molecules possibly present in the 0.1 mol L^‑1^ Li_2_SO_4_.


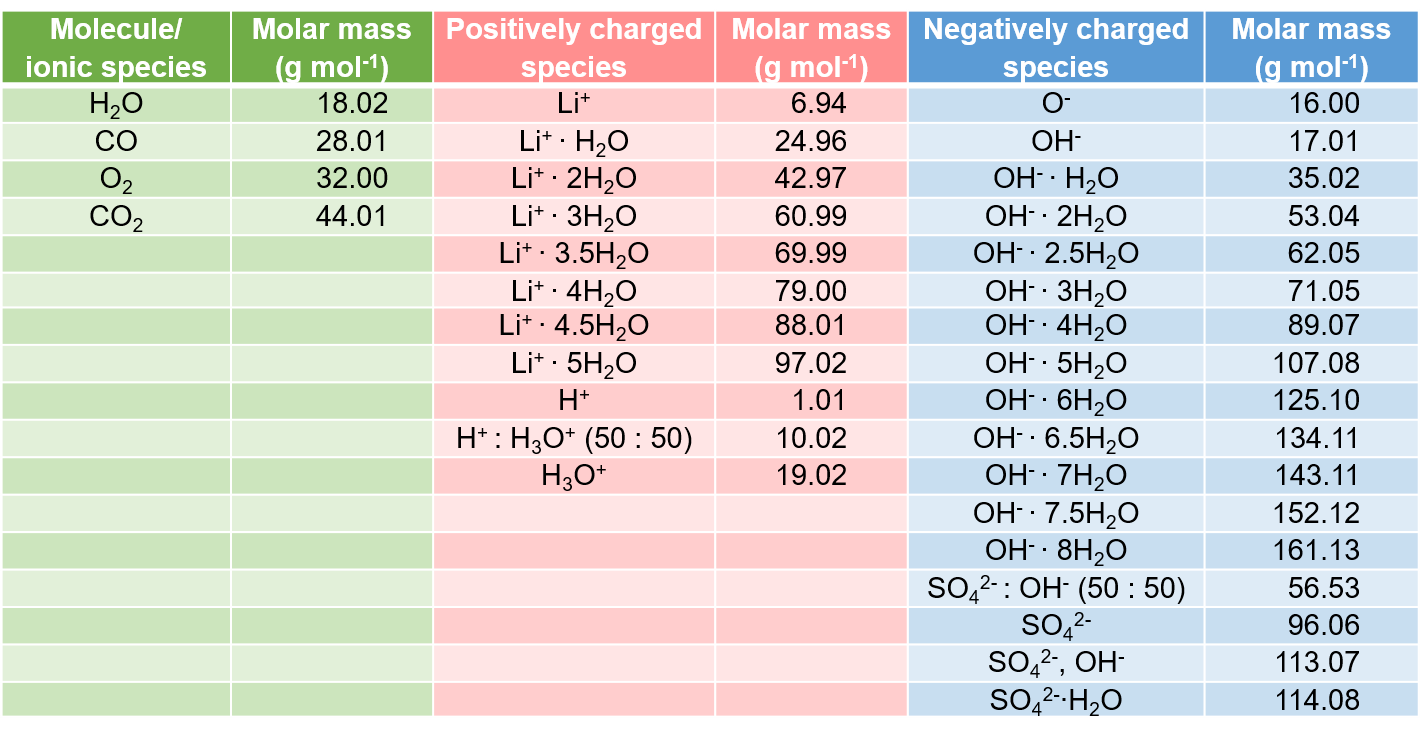


# D_2_O as a solvent

Furthermore, D_2_O solvent (*ε* = 60; 1.87 D) was used for LiNO_3_ salt to see its influence on EDL formation compared to the water molecule (*ε* = 80; 1.84 D). D_2_O molecule has lower hydration affinity (*ε*), as it does not create hydrogen bonds as easily as a H_2_O molecule. Moreover, D_2_O molecule is slightly more polar than H_2_O and denser, therefore can affect EDL at the electrode/electrolyte interface, leading to a narrower PZC region. If min. specific capacitance will be considered as one value, it divides PZC region in half. However, to the best of our knowledge, the PZC region discussed here informs about the perm-selectivity failure of electrode material in contact with a specific liquid electrolyte. Therefore, as discussed in the literature, this region should be excluded from ion flux divagation. In the further section, we will prove this and explain the implication of incorrectly assumed PZC value on the charge storage mechanism description.

# References:

1 N. Shpigel, M.D. Levi, S. Sigalov, O. Girshevitz, D. Aurbach, L. Daikhin, P. Pikma, M. Marandi, A. Jänes, E. Lust, N. Jäckel, V. Presser, In situ hydrodynamic spectroscopy for structure characterization of porous energy storage electrodes, Nature materials 15(5) (2016) 570-575.

2 S. Boyd, K. Ganeshan, W.-Y. Tsai, T. Wu, S. Saeed, D.-e. Jiang, N. Balke, A.C.T. van Duin, V. Augustyn, Effects of interlayer confinement and hydration on capacitive charge storage in birnessite, Nature materials 20(12) (2021) 1689-1694.

3 P. Simon, Y. Gogotsi, Perspectives for electrochemical capacitors and related devices, Nature materials 19(11) (2020) 1151-1163.

4 Alexander C. Forse, John M. Griffin, C. Merlet, J. Carretero-Gonzalez, A.-Rahman O. Raji, Nicole M. Trease, Clare P. Grey, Direct observation of ion dynamics in supercapacitor electrodes using in situ diffusion NMR spectroscopy, Nature Energy 2(3) (2017).

5 F.W. Richey, B. Dyatkin, Y. Gogotsi, Y.A. Elabd, Ion Dynamics in Porous Carbon Electrodes in Supercapacitors Using in Situ Infrared Spectroelectrochemistry, Journal of the American Chemical Society 135(34) (2013) 12818-12826.

6 A. Ghosh, Y.H. Lee, Carbon-Based Electrochemical Capacitors, ChemSusChem 5(3) (2012) 480-499.

7 H. Shao, Y.-C. Wu, Z. Lin, P.-L. Taberna, P. Simon, Nanoporous carbon for electrochemical capacitive energy storage, Chemical Society reviews 49(1) (2020) 35-339.

8 A. Platek-Mielczarek, E. Frackowiak, K. Fic, Specific carbon/iodide interactions in electrochemical capacitors monitored by EQCM technique, Energy & environmental science 14(4) (2021) 2381-2393.

9 L. Borchardt, M. Oschatz, S. Kaskel, Tailoring porosity in carbon materials for supercapacitor applications, Materials horizons 1(2) (2014) 157-168.

10 H. Jiang, P.S. Lee, C. Li, 3D carbon based nanostructures for advanced supercapacitors, Energy & environmental science 6(1) (2012) 41-53.

11 G. Wang, L. Zhang, J. Zhang, A review of electrode materials for electrochemical supercapacitors, Chemical Society reviews 41(2) (2012) 797-828.

12 H. Yin, H. Shao, B. Daffos, P.-L. Taberna, P. Simon, The effects of local graphitization on the charging mechanisms of microporous carbon supercapacitor electrodes, Electrochemistry communications 137 (2022) 107258.

13 P. Bujewska, B. Gorska, K. Fic, Redox activity of selenocyanate anion in electrochemical capacitor application, Synthetic metals 253 (2019) 62-72.

14 D. Gastol, J. Walkowiak, K. Fic, E. Frackowiak, Enhancement of the carbon electrode capacitance by brominated hydroquinones, Journal of power sources 326 (2016) 587-594.

15 J. Wu, Y. Ben, D. Battigelli, H.-C. Chang, Long-Range AC Electroosmotic Trapping and Detection of Bioparticles, Industrial & engineering chemistry research 44(8) (2005) 2815-2822.

16 J.P. Guyer, An Introduction to Electrochemical Impedance Assessment of Coatings, The Clubhouse Press, El Macero, California, 2019.

17 B.E. Conway, Electrochemical Supercapacitors Scientific Fundamentals and Technological Applications / by B. E. Conway, 1st 1999. ed., Springer US, New York, NY, 1999.

18 M.F. Dupont, S.W. Donne, A Step Potential Electrochemical Spectroscopy Analysis of Electrochemical Capacitor Electrode Performance, Electrochimica acta 167 (2015) 268-277.

19 A. Platek, J. Piwek, K. Fic, E. Frackowiak, Ageing mechanisms in electrochemical capacitors with aqueous redox-active electrolytes, Electrochimica acta 311 (2019) 211-220.
